# A new image-based tool for the high throughput phenotyping of pollen viability: evaluation of inter- and intra-cultivar diversity in grapevine

**DOI:** 10.1186/s13007-017-0267-2

**Published:** 2018-01-09

**Authors:** Javier Tello, María Ignacia Montemayor, Astrid Forneck, Javier Ibáñez

**Affiliations:** 1 Instituto de Ciencias de la Vid y del Vino (CSIC, Gobierno de La Rioja, Universidad de La Rioja), Carretera de Burgos km 6, Finca La Grajera, 26007 Logroño, Spain; 2 Division of Viticulture and Pomology, Department of Crop Sciences, University of Natural Resources and Life Sciences Vienna (BOKU), Konrad Lorenz Straße 24, 3430 Tulln, Austria

**Keywords:** Computer vision, Plant phenotyping tool, Pollen sterility, Pollination, *Vitis vinifera* L.

## Abstract

**Background:**

Low pollen viability may limit grapevine yield under certain conditions, causing relevant economic losses to grape-growers. It is usually evaluated by the quantification of the number of viable and non-viable pollen grains that are present in a sample after an adequate pollen grain staining procedure. Although the manual counting of both types of grains is the simplest and most sensitive approach, it is a laborious and time-demanding process. In this regard, novel image-based approaches can assist in the objective, accurate and cost-effective phenotyping of this trait.

**Results:**

Here, we introduce PollenCounter, an open-source macro implemented as a customizable Fiji tool for the high-throughput phenotyping of pollen viability. This tool splits RGB images of stained pollen grains into its primary channels, retaining red and green color fractionated images (which contain information on total and only viable pollen grains, respectively) for the subsequent isolation and counting of the regions of interest (pollen grains). This framework was successfully used for the analysis of pollen viability of a high number of samples collected in a large collection of grapevine cultivars. Results revealed a great genetic variability, from cultivars having very low pollen viability (like Corinto Bianco; viability: 14.1 ± 1.3%) to others with a very low presence of sterile pollen grains (Cuelga; viability: 98.2 ± 0.5%). A wide range of variability was also observed among several clones of cv. Tempranillo Tinto (from 97.9 ± 0.9 to 60.6 ± 5.9%, in the first season). Interestingly, the evaluation of this trait in a second season revealed differential genotype-specific sensitivity to environment.

**Conclusions:**

The use of PollenCounter is expected to aid in different areas, including genetics research studies, crop improvement and breeding strategies that need of fast, precise and accurate results. Considering its flexibility, it can be used not only in grapevine, but also in other species showing a differential staining of viable and non-viable pollen grains. The wide phenotypic diversity observed at a species level, together with the identification of specific cultivars and clones largely differing in this trait, pave the way of further analyses aimed to understand the physiological and genetic causes driving to male sterility in grapevine.

**Electronic supplementary material:**

The online version of this article (10.1186/s13007-017-0267-2) contains supplementary material, which is available to authorized users.

## Background

The commercial production of wine and table grapes (*Vitis vinifera* L.) is mostly based on the cultivation of plants with hermaphroditic flowers, in which self-fertilization is the major route for pollination. Fruit-set rate (i.e. proportion of ovaries in an inflorescence that become fruits) can be highly variable in grapevine [1], and it is generally considered as normal if greater than 50%, and insufficient if less than 30% [2]. In fact, poor fruit-set is said to limit crop yield under certain conditions, causing relevant economic losses to grape-growers [1]. Fruit-set is affected by many factors, including climate (e.g. light, temperature, rain), vineyard management (e.g. pruning system, row configuration, training system) and genetic factors [3–7]. Regarding the latter, low pollen viability [5, 8], low ovule fertility [6], low germination rate [9], or the development of anomalous flowers [10] are some of the reasons explaining poor fruit-set and the excessive abscission of flowers (termed shatter, shedding or by the French word “coulure”) [11].

Grapevine pollen formation and development follow a time-course process [12]. Under optimal climatic conditions, sporogenous cells can be detected in the anthers as soon as inflorescences are clearly visible in the growing shoot. Meiosis takes place after some days, giving rise to tetrads which rapidly release microspores in the loculus. Microspores vacuolate thereafter, and pollen mitosis take place just before anthesis, when pollen grains are mature enough to be released from the anthers [13, 14]. In this regard, genotype-specific differences in pollen starch reserves affect this developmental process, having an effect in the successful obtaining of fully mature pollen grains at anthesis [13]. Additionally, pollen production is highly variable between grapevine cultivars. In a work performed in a set of table grape cultivars, Kelen et al. [7] report a variation ranging between ca. 3000 and 9000 pollen grains per flower. These figures suggest that pollination and fruit set might be ensured even at very low rates of pollen viability. Nevertheless, pollen sterility may be accompanied by a certain degree of ovule sterility [15], becoming a more limiting factor for successful berry set and subsequent grape production, since there are only four ovules per flower [11], and at least one fertilized ovule is needed for a normal berry set.

Pollen viability indicates the ability of the pollen grain to deliver sperm cells to the embryo sac following compatible pollination [16]. Consequently, it can be considered as a measurement of pollen grain quality and vigor [17]. In this regard, a positive relationship between pollen viability and pollen germination capability has been suggested [7], and Royo et al. [18] have recently reported the incapability of the non-viable Corinto Bianco grapevine pollen grains to germinate in vitro, indicating that viable pollen is needed for a successful germination and so for seed and fruit development and growth. Pollen viability has been studied by different methods, including the evaluation of seed- and fruit-set, diverse in vitro and in vivo germination tests, and by different staining techniques. Alexander’s staining [19] is one of the most common methods, since it allows the distinction between viable and non-viable (sterile, aborted) pollen grains based on the differential staining of the pollen protoplasm and the cellulose contained in pollen walls. The method has been suggested to be appropriate for large scale breeding programs, as it correlates with pollen germination rates and it is simple and easy to use [20]. After pollen grains staining, viability can be rated by the manual (eye) counting of non-viable (stained in pale turquoise blue) and viable (stained dark blue or purple) pollen grains. Although this manual approach is the simplest and most sensitive one to evaluate pollen viability, it is a slow and laborious process that might result in diminished accuracy and precision (reproducibility) rates. In an effort to automate this task, Kelly et al. [21] suggested the use of pollen grain size variation as an indirect evaluation of pollen viability, considering that viable grains are considerably bigger than non-viable grains. Authors found a significant positive correlation between the proportion of viable and non-viable grains (after differential staining), and the so-called pollen size index (PSI), which relativizes the number of pollen grains above a certain size by the total number of grains. This approach has been used for the automatic estimation of pollen viability in species like *Mimulus guttatus*, *Collinsia verna* and *Beta vulgaris* [21, 22].

Image-based phenotyping is revolutionizing many areas of plant research [23]. The use of high-throughput systems is being used to extract biological information from image data for subsequent decision support. The analysis of microscope preparations can be sped-up by the collection of color digital microscope images (RGB images) for their further automate processing with specific image analysis systems [24]. As an example, an image processing workflow for the automated analysis of images of stained sections of maize internodes has been recently proposed to discriminate and quantify lignified and non-lignified tissues [25]. In this regard, the development of objective, accurate and rapid cost-effective methods for the high-throughput evaluation of pollen viability will be helpful in different palynological areas, including genetics research, crop improvement and breeding studies [17]. In addition, it will be useful to evaluate the effect of storage on pollen viability and longevity in genebanks [26], and to assess the effect of different environmental factors on pollen performance [27]. Although image based technologies have been already applied in palynological analyses [28–30], their use has been mainly restricted to the automatic counting of pollen grains of several plant species, mostly for their morphological classification according to predefined categories.

Here, we introduce a customizable macro developed for the open-source Fiji image-analysis platform [31] for the automatic analysis of digital microscope RGB images of pollen grains after Alexander’s modified staining [32], as a solution to enable an automated and fast pollen viability phenotyping. This macro, PollenCounter, splits digital images into its primary channels, and isolates the regions of interest (pollen grains) from the background in the red and green color fractionated images to address the independent counting of total and viable pollen grains. The PollenCounter macro has been designed and validated in a sample of grapevine cultivars that show a high degree of pollen viability variability, providing accurate and precise information in all the tested scenarios. In addition, it has been applied to evaluate if there is some difference in pollen viability within different inflorescences of the same clone, as well as to show the range of variability among different clones of cv. Tempranillo Tinto and a high number of table- and wine-grape cultivars.

## Methods

### Plant material

#### Grapevine cultivars and flowers sampling

In this study, 120 grapevine accessions have been analyzed (Additional file 1). These accessions belong to four different entities, and they were studied on their own experimental site: (I) the Grapevine Germplasm Collection of the Instituto de Ciencias de la Vid y del Vino (ICVV; FAO Institute Code ESP-217), in Logroño (La Rioja, Spain); (II) the Experimental Grapevine Collection of the Universität für Bodenkultur Wien (BOKU), in Tulln an der Donau (Niederösterreich, Austria); (III) the cv. Tempranillo Tinto clones collection of a commercial grapevine nursery (VP, Viveros Provedo S.A.), also in Logroño; and (IV) a private vineyard of cv. Tempranillo Blanco (FZ), in Fonzaleche (La Rioja, Spain). Accessions from BOKU were used to evaluate sampling effect, accessions from ICVV were used to evaluate the inter-cultivar genetic diversity, and Tempranillo Tinto clones from VP were used to evaluate the intra-cultivar genetic variability. In these four plots, plants are maintained following standard conditions. Cultivar identity was confirmed by SSRs and/or SNPs analyses [33]. If available, cultivar prime name according to the VIVC database (http://www.vivc.de) has been used in the manuscript.

##### BOKU sampling

To evaluate within-clone differences in pollen viability, flowers from the basal and second-order inflorescences from the proximal and distal shoots were collected early in the morning in three plants of the same clone. The analysis was done one season (2017) in four wine cultivars (Cabernet Franc, Chardonnay, Gewuerztraminer and Sauvignon Blanc) (Additional file 1). In BOKU plot, plants are trained as a Guyot system with a single cane of 8 buds and one spur of 2 retained buds.

##### ICVV, FZ and VP sampling

For the inter- and intra-cultivar analyses of pollen viability variability, the first inflorescences from a random shoot from two plants were sampled early in the morning at ICVV, VP and FZ plots in 2015. This study involved 116 grapevine accessions (111 in ICVV, 4 in VP and 1 in FZ). This analysis was repeated in 2017 on 24 selected varieties (ICVV) and on Tempranillo Tinto clones (VP) (Additional file 1). In ICVV, VP and FZ plots, plants are trained on a Royat system, composed of a double spur-pruned permanent cordon with 6 spurs of 2 buds per vine.

#### Pollen sample preparation and imaging

**Fig. 1:**
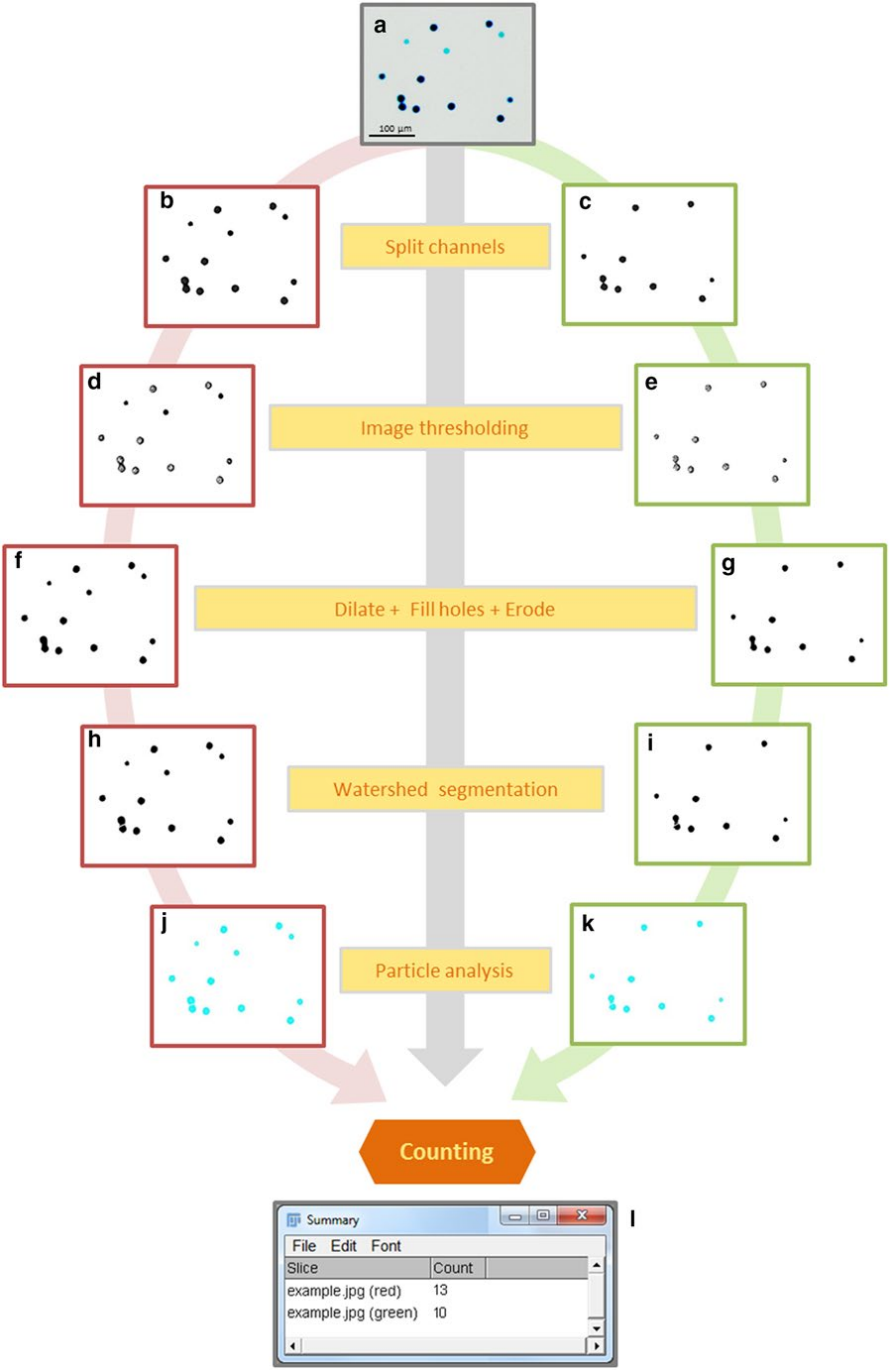
PollenCounter pipeline for the automated detection and counting of total and viable pollen grains. In **a**, an input RGB image of grapevine pollen grains after Alexander’s modified staining is shown (dark pollen grains are viable, whilst pale grains are sterile). This image is firstly segmented into its primary colors, and the color fractionated grayscale pictures for the red (**b**) and green (**c**) channels are taken for their parallel analysis (red and green image frame lines, respectively). Images are then segmented into black regions of interest (pollen grains) and a homogenous white background (**d**, **e**). Regions of interest are closed and filled with black pixels (**f**, **g**) before the use of a watershed algorithm for the separation of touching regions of interest (**h**, **i**). Identified pollen grains are then counted, and output images (**j**, **k**) are automatically saved as.jpg files. Note that in these images, counted pollen grains are automatically colored in cyan. In **l**, an example of the output pivot table with the counting results in both channels is shown. The main commands implemented in PollenCounter are indicated in yellowish boxes (grey arrow). For simplification, only a representative area of one of the processed images from cv. Vermentino is presented

To determine pollen viability, inflorescences were collected at full bloom (modified E-L 23 stage [34]), when 50% of flower caps have fallen. After collection, they were transported to the laboratory and maintained at room temperature until their analysis, which was finished within the same day. Recently opened flowers (with erect stamen filaments and light yellowish anthers) from the central section of the inflorescence were then collected and stained following Alexander’s modified staining [32], which can differentiate between aborted (non-viable) and non-aborted (viable) grains (Fig. 1a). Briefly, three-four flowers were selected, soaked in 40 μL of staining solution and shaken vigorously for 15 s to facilitate pollen release. 20 μL of the solution were then transferred onto a pre-heated microscope slide and observed to take digital images. Samples from ICVV, FZ and VP samples were observed using a Zeiss SteREO Discovery V20 stereo-microscope, and images were taken using a Zeiss AxioCam camera and AxioVision software (v. 4.8, Zeiss). Samples from BOKU were visualized with an Olympus CX41 microscope equipped with an Olympus SC50 digital camera using CellSens Entry software (v. 1.14, Olympus). Image contrast and saturation was adapted to maximize the differentiation between aborted and non-aborted pollen grains. In total, 4126 RGB images saved as JPEG files with an average number of 130 pollen grains per image were taken for further analysis.

### Automated analysis of pollen images for pollen grains counting

The workflow designed for the automatic counting of viable and non-viable pollen grains is shown in Fig. 1. The open-source Fiji platform [31] was used for macro development and subsequent image analysis. It is based on the popular open-source software focused on biological-image processing ImageJ v. 1.51 (US National Institutes of Health). This sequential analysis was executed in batch mode on the entire set of images stored in a predefined directory. Briefly, each input RGB image is split into its three primary color channels (red, green and blue) as color-fractionated grayscale pictures. Considering that they hold independent information regarding total and viable pollen grains (Fig. 1b, c), only red and green channels are retained for their analysis. The threshold of both color-channel images is automatically set to segment images into background and individual regions of interest (in our case, pollen grains), and every image is black and white-converted. As a result, images with pollen grains transformed to black regions of interest and a homogenous white background are obtained (Fig. 1d, e). Next, the Dilate and Fill holes operations are applied to generate solid particles, by adding black pixels to the edges and inner sections of the black regions of interest. Additional pixels from the edges are then removed using the Erode operation (Fig. 1f, g). The automatic separation of touching regions is performed with a watershed segmentation algorithm. It calculates the Euclidean distance map and looks for the ultimate eroded points, which are then dilated as far as possible (either until the edge of the particle is reached, or it touches another region of interest) (Fig. 1h, i). Regions of interest were then measured (number, surface areas and diameters) using the Analyze Particles command (Fig. 1j,k). In this regard, only particles with a surface area ranging between 60 and 800 pixels^2^ were counted in the red-channel images (Pollen grains_R_), whereas only those with a surface area between 100 and 800 pixels^2^ were considered in the green-channel images (Pollen grains_G_). For both channels, circularity (calculated from diameter data) range was set to 0.40–1.00. After processing, automatic counting results are shown in a pivot table that can be saved by user as a .txt or .xls file (Fig. 1l). Additionally, two.JPEG files corresponding to the analysis of red and green channels are created in the directory. In these images, the detected and counted pollen grains are shown in cyan and the discarded structures in black.

The custom Fiji macro for the automatic counting of total and viable pollen grains, called PollenCounter, and a set of images are available as Additional files 2 and 3.

### Evaluation and validation of the automatic approach

For the validation of the novel approach, a subset of 392 RGB images from 19 cultivars grown in ICVV and FZ plots (Albillo Real, Bouschet Petit, Cuelga, Dominga, Gamay noir, Gewuerztraminer, Mencía, Molar, Pinot Noir, Planta Mula, Ruby Cabernet, Rubired, Sauvignon Blanc, Schiava Grossa, Silvaner Gruen, Syrah, Tempranillo Blanco, Trousseau Noir and Vijiriega Común) were chosen. These images were specifically selected for their variable number of pollen grains (from 20 to 1042 pollen grains) and viability rate (from ca. 20 to 100%).

#### Manual analysis of pollen images for viability estimation

The number of viable and non-viable pollen grains was manually counted in each image considering their differential staining (pale blue = non-viable/dark blue = viable). Manual pollen viability per image [MPV_img_ (%)] was calculated as follows:

$$MPV_{img}\, \left( \% \right) = \frac{Dark\,blue\,pollen\,grains}{All\,pollen\,grains} \times 100$$

#### Automatic analysis of pollen images for viability estimation

Automatic pollen viability per image [APV_img_ (%)] was calculated using the information obtained with the automatic approach as follows:

$$APV_{img}\, \left( \% \right) = \frac{{Pollen\,grains_{G} }}{{Pollen\,grains_{R} }} \times 100$$

#### Pollen size index (PSI) determination

As suggested by Kelly et al. [21], we calculated the pollen size index per image (PSI_img_) in a subsample of 38 images from the 19 previously listed cultivars (2 images per cultivar). To this aim, the surface area of all pollen grains in each image was individually obtained (in pixels^2^) using the Analyze Particles command of Fiji, and viable and non-viable pollen grain surface areas were compared to determine the optimal surface area threshold capable to differentiate both types (Additional file 4A). Although the overlapping observed between the distribution of the area of viable and non-viable pollen grains suggested that this method would not be useful in grapevine, a general threshold of 118 pixels^2^ was established for PSI (%) calculation. This value corresponds to the average value of the area of the smallest viable pollen grain detected in each of the 38 images. PSI (%) was calculated as follows:

$$PSI_{img}\, \left( \% \right) = \frac{{Pollen\,grains\,over\,118\,pixels^{2} }}{Total\,pollen\,grains} \times 100$$

### Grapevine pollen viability assessment

To estimate pollen viability in a sample (inflorescence), automatic pollen viability [APV (%)] was calculated as follows:

$$APV\,\left( \% \right) = \frac{{\sum Pollen\,grains_{G} }}{{\sum Pollen\,grains_{R} }} \times 100,$$

where ∑Pollen grains_G_ and ∑Pollen grains_R_ indicate the sum of automatically-counted pollen grains in all the green- and red-channel images taken from a sample, respectively. In average, more than 1000 pollen grains per sample were analyzed.

### Statistical analysis

The performance of the automatic method was evaluated by comparing the automatic results (total counting and viable counting) with those manually obtained (n = 392 images) through linear regression models and associated coefficients of determination (R^2^). In addition, Bland–Altman plots were calculated as previously indicated [35] to estimate the accuracy of both approaches. Box-plots combined with independent t-tests were used to detect significant differences (*p* ≤ 0.05) between pollen viability (%) rates obtained manually [MPV_img_ (%)] and by the automatic method [APV_img_ (%)]. The performance of the PSI method was evaluated by comparing the automatic result [PSI_img_ (%)] with the manual value [MPV_img_ (%)], through a linear regression model and its associated coefficient of determination (R^2^) (n = 38 images).

To analyze within-clone differences in APV (%), BOKU samples were used (n = 384 images). First, a one-way ANOVA was individually calculated for each cultivar to evaluate if APV (%) varied significantly (*p* ≤ 0.05) between plants of the same clone (cultivar). Then, a three-way ANOVA with Fisher’s LSD post hoc tests was calculated in the whole set of samples to check if any of the three factors considered (genotype, shoot position and inflorescence order) played a significant effect (*p* ≤ 0.05) on APV (%). For this analysis, four levels were considered for genotype (Cabernet Franc, Chardonnay, Gewuerztraminer and Sauvignon Blanc), two for shoot position (proximal and distal) and two for inflorescence position (first and second order) factors.

A Fisher’s LSD-test was performed to assess differences in APV (%) between Tempranillo Tinto clones, being considered significant at *p* ≤ 0.05.

The normality of the data was graphically assessed using histograms. All calculations were done using SPSS v. 21.0 (IBM, Chicago, IL, USA).

## Results

### Automatic pollen counting tool designing and validation

**Fig. 2:**
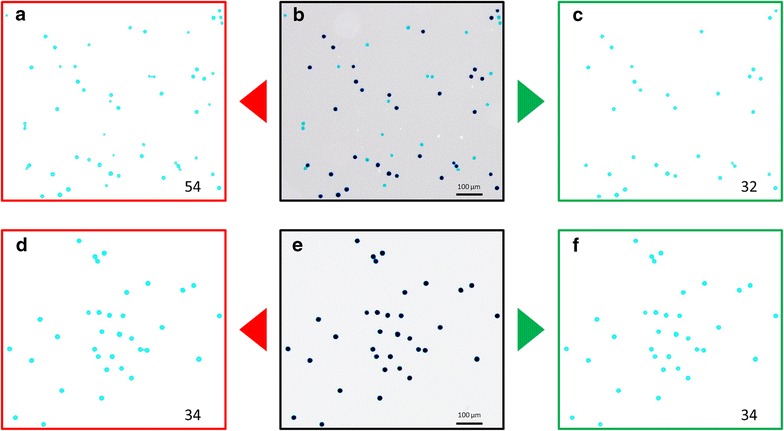
Original and processed images obtained for two typical pollen samples with low (**a**–**c**, cv. Albillo Real) and high (**d**–**f**, cv. Forcallat Tinta) viability. Original images are shown in **b** and **e**. Images obtained in the red (**a**, **d**) and green (**c**, **f**) channels after PollenCounter use are shown with the number of pollen grains automatically identified (lower right corners). For simplification, only representative areas of the processed images are shown

Two typical RGB images of two cultivars with low (cv. Albillo Real) and high (cv. Forcallat Tinta) pollen viability rates after Alexander’s modified staining approach [32] are shown in Fig. 2b, e. As previously reported [21, 22], a difference in size between viable and non-viable pollen grains was observed for the different cultivars evaluated (see Figs. 1a, 2b and Additional file 5 for some examples), with non-viable pollen grains slightly smaller. Nonetheless, the calculation of PSI_img_ yield a non-significant correlation with the manual evaluation of pollen viability (MPV_img_; R^2^ = 0.15; *p* > 0.01, Additional file 4B) in a subsample of 38 images of 19 grapevine cultivars, and so its usefulness is limited when considering multi-cultivar genetic frameworks.

On the other hand, viable and non-viable pollen grains can be easily identified according to their differential staining in samples with both types of pollen grains (Figs. 1a, 2b and Additional file 5). The three dyes used in the Alexander’s modified staining solution (malachite green, acid fuchsin and orange G) split into very different red, green and blue image profiles (see Additional file 6). In this regard, it is easy to identify malachite green dye (which stains cellulose in pollen walls, present in all pollen grains) in the red channel, as happens with acid fuchsin dye (which stains pollen protoplasm, only present in mature viable pollen grains) in the green one (Additional file 5). Orange G (clearly identified in the blue channel) reinforces the differentiation of the other two dyes, aiding in the distinction of viable and non-viable pollen grains. These differences pave the way to test the use of the red and green channels for the automatic differentiation and counting of total and viable pollen grains through an image-based approach. Consequently, we developed a processing tool, called PollenCounter, to be used in the open-source Fiji platform for the segmentation of the three channels in our set of images, retaining green and red layers (Fig. 2) for their independent analysis, as detailed in the pipeline indicated in “Methods” (Fig. 1).

The use of PollenCounter allowed the differential counting of total and viable pollen grains with absolute reproducibility while substantially reducing the time needed for the analysis. Considering the subset of 392 images, an average of 240 s of dedicated work were needed per image for the manual counting of viable and non-viable pollen grains, whereas this time was reduced to 3.5 s when PollenCounter was run in an IntelCore i3 laptop.

**Fig. 3:**
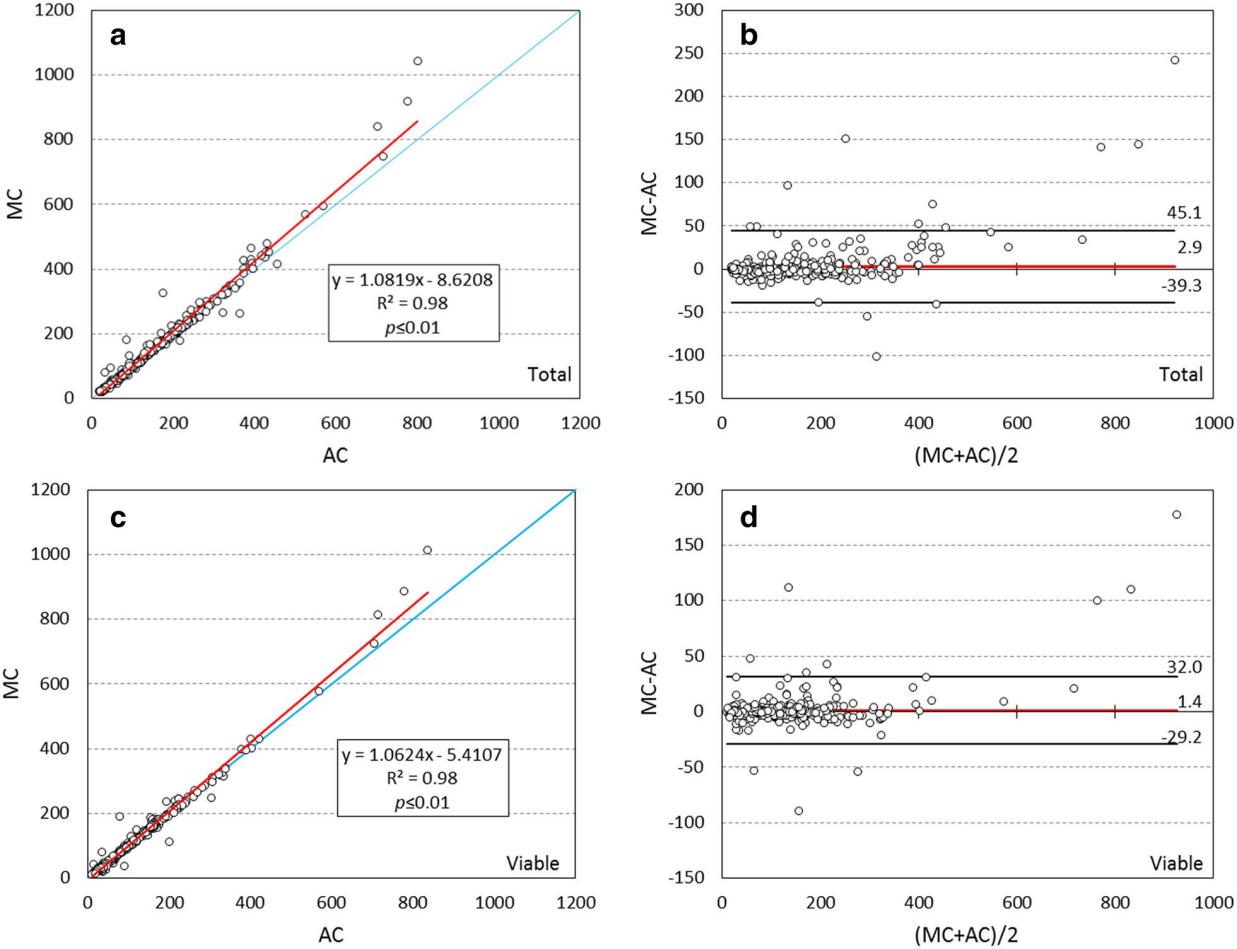
Correlation analyses (**a**, **c**) and Bland–Altman plots (**b**, **d**) comparing manual (MC) and automatic (AC) counting of total (**a**, **b**) and viable (**c**, **d**) pollen grains. In **a** and **c**, red solid lines shows linear regression line, whereas the identity line (x = y) is shown as a blue solid line. In **b** and **d**, red solid lines indicate the mean difference between both approaches, while upper and lower solid black lines represent limits of agreement

PollenCounter accuracy was evaluated by comparing the number of total and viable pollen grains counted manually and automatically in the individual images taken from 19 cultivars (n = 392, Fig. 3). A significant correlation between manual and automatic values (R^2^ = 0.98, *p* ≤ 0.01) was obtained both for total and viable pollen grains, and regression lines nearly match identity lines (x = y) (Fig. 3a, c). Similarly, the Bland and Altman approach showed a high agreement between the automatic and manual number of total and viable pollen grains, with a mean difference of 2.9 and 1.4 pollen grains between both systems, respectively (Fig. 3b, d). The individual differences between these two counting systems were well distributed within the interval limits and no bias was observed, especially in the range between 30 and 284 pollen grains, which constitute percentile 10 and 90 of the whole set of images analyzed, respectively. Moreover, 95% confidence intervals were small enough to sustain that the automatic method could substitute the manual one. Our approach also yield high correlation results (R^2^ = 0.98; *p* ≤ 0.01) between APV_img_ (%) estimations and MPV_img_ (%) values in the same subsample of images (n = 38) used for testing the usefulness of the pollen size index (Additional file 4C).

**Fig. 4:**
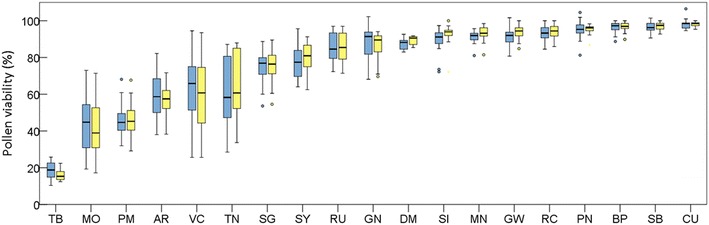
Viability rates (%) obtained using automatic (APVimg, blue) and manual (MPVimg, yellow) pollen grain counting in 19 grapevine cultivars (AR: Albillo Real; BP: Bouschet Petit; CU: Cuelga; DM: Dominga; GN: Gamay noir; GW: Gewuerztraminer; MN: Mencia; MO: Molar; PM: Planta Mula; PN: Pinot Noir; RC: Ruby Cabernet; RU: Rubired; SB: Sauvignon Blanc; SG: Schiava Grossa; SI: Silvaner Gruen; SY: Syrah; TB: Tempranillo Blanco; TN: Trousseau Noir; VC: Vijiriega Común). No significant differences between manual and automatic approaches were observed for any cultivar (*t* test, *p* > 0.05). Whiskers indicate standard deviations between images

On a next step, the validity of PollenCounter to estimate pollen viability was checked in the 19 cultivars to evaluate potential genotype-specific inconsistencies. In this regard, APV_img_ (%) and MPV_img_ (%) values were compared for each cultivar independently, using the information obtained from each processed image. No significant differences were found on pollen viability rates between the manual and the automatic approach in any of the grape cultivars (Fig. 4), even when analyzing genotypes with very low (e.g. Tempranillo Blanco) or very high (e.g. Cuelga) pollen viability.

As a whole, PollenCounter provided precise, accurate and valid counts of total and viable grains from pollen samples stained using Alexander’s protocol, which could be used to estimate pollen viability rate in diverse grapevine cultivars. Consequently, pollen viability results presented in the next sections have been obtained using PollenCounter.

### Evaluation of between- and within-vines pollen viability differences

**Fig. 5:**
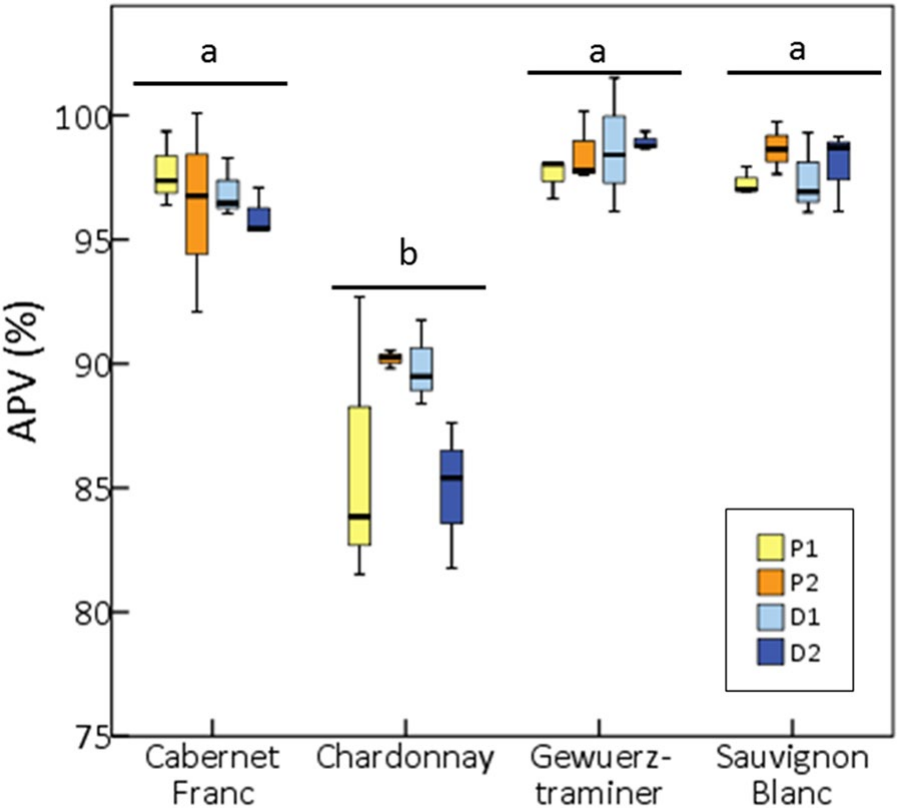
Automatic pollen viability rates [APV (%)] obtained in different inflorescences of four different grapevine cultivars with PollenCounter. Different lowercase letters indicate significant differences between cultivars (*p* ≤ 0.05). P1: First inflorescence of the proximal shoot (yellow); P2: Second inflorescence of the proximal shoot (orange); D1: First inflorescence of the distal shoot (light blue); D2: second inflorescence of the distal shoot (dark blue). Whiskers indicate standard deviations between inflorescences

One way ANOVA revealed no significant differences in APV (%) between the three plants sampled for any of the cultivars assayed: Cabernet Franc (*p* = 0.10), Chardonnay (*p* = 0.16), Gewuerztraminer (*p* = 0.09) and Sauvignon Blanc (*p* = 0.81). In a more detailed evaluation, we tested the effect of shoot position (proximal/distal) and inflorescence order (first/second) on APV (%). The three-way ANOVA indicated that genotype is the only factor contributing significantly to variability (F = 45.16; *p* ≤ 0.05), and no significant effect was released for shoot position (F = 0.06; *p* = 0.80) nor inflorescence order (F = 0.25; *p* = 0.62). Similarly, no significant two-way or three-way interactions for the factors analyzed were obtained. Additional Fisher’s LSD post hoc tests revealed a significant (*p* ≤ 0.05) lower APV (%) values for Chardonnay inflorescences (in average, 88.4 ± 3.9%) compared to the other three cultivars (96.7 ± 1.9, 98.0 ± 1.5 and 98.5 ± 1.2% for Cabernet Franc, Gewuerztraminer and Sauvignon Blanc inflorescences, respectively), but no significant differences between any of the inflorescences of the same genotype were found (*p* > 0.05) (Fig. 5).

### Pollen viability in grapevine clones and cultivars

**Fig. 6:**
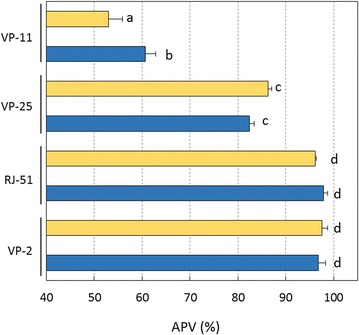
Automatic pollen viability rates [APV (%)] obtained for four Tempranillo clones (VP-11, VP-25, RJ-51, VP-2) in two different seasons—2015 (blue bars) and 2017 (yellow bars)—with PollenCounter. Different lowercase letters indicate significant differences between samples (*p* ≤ 0.05). Whiskers indicate standard deviations between inflorescences

On a next level of comparison, APV (%) values from 4 different clones of the same cultivar (Tempranillo Tinto) sampled in the same experimental plot (VP) were compared (Fig. 6). We found two Tempranillo clones with very high APV (%) values (RJ-51 and VP-2, > 90%), one clone with high APV (%) (VP-25, 75–90%), and one clone with medium APV (%) (VP-11, 50–75%). These APV (%) values were rather stable considering 2015 and 2017 seasons.

Next, we compared pollen viability between different cultivars. Table 1 indicates the average and standard deviation of APV (%) obtained for the 111 accessions (107 cultivars) analyzed from the ICVV Grapevine Germplasm Collection in 2015 using PollenCounter. In general, we found high average APV (%) values for the cultivars analyzed in this work, with 81.0% of individuals (90 accessions) over 75%, and 49.5% (55 accessions) over 90%. In contrast, the lowest pollen viability value was found for the parthenocarpic cultivar Corinto Bianco, with an APV (%) of 14.1 ± 1.3%. In addition, we found other five accessions with average pollen viability lower than 50%: the interspecific hybrid Flot Rouge (35.1 ± 11.1), and the *V. vinifera* cultivars Planta Mula (44.9 ± 6.5), Molar (45.4 ± 9.8), Tempranillo Tinto (49.9 ± 0.8) and Vermentino (49.9 ± 3.2). Considering that they were grown under similar conditions in the same location and plot, the wide range of diversity observed between cultivars evidences a clear genetic effect on pollen formation and/or development, driving to different pollen viability rates. Additionally, no effect of the pruning system (Guyot vs. Royat) on flower vigor and pollen viability was observed when both systems were used for a same cultivar (Cabernet Franc, Chardonnay, Gewuerztraminer and Sauvignon Blanc), although they were tested in two different locations and two different clones (Fig. 5 and Table 1).

**Table 1:** List of grapevine accessions (n = 111), corresponding to 107 different cultivars, sampled in the ICVV Grapevine Germplasm Collection for this study

Plot code	Cultivar name	Main use	Grape skin color	Pollen viability (APV, %)	Pollen viability rate
01GRAJ007E	Corinto Bianco	W	Green yellow	14.15 ± 1.28	Very low
01GRAJ018K	Flot Rouge	W	Blue black	35.10 ± 11.05	Low
01GRAJ010A	Planta Mula	W/T	Blue black	44.87 ± 6.53	Low
01GRAJ022H	Molar	W	Blue black	45.42 ± 9.82	Low
01GRAJ021K	Tempranillo Tinto	W/T	Blue black	49.93 ± 0.79	Low
01GRAJ015L	Vermentino	W/T	Green yellow	49.94 ± 3.16	Low
01GRAJ012D	Garganega	W/T	Green yellow	52.90 ± 3.59	Medium
01GRAJ015D	Tempranillo Blanco	W/T	Green yellow	57.26 ± 6.89	Medium
01GRAJ020H	Mollar Cano	W/T	Blue black	57.89 ± 1.77	Medium
01GRAJ015H	Trebbiano Toscano	W	Green yellow	58.55 ± 14.91	Medium
01GRAJ010F	Albillo Real	W	Green yellow	60.38 ± 9.67	Medium
01GRAJ013L	Muscat a Petits Grains Blancs	W/T	Green yellow	61.9 ± 17.47	Medium
01GRAJ019G	Trousseau Noir	W	Dark red violet	62.95 ± 24.9	Medium
01GRAJ011J	Vijiriega Común	W	Green yellow	65.52 ± 0.82	Medium
01GRAJ014D	Parellada	W	Green yellow	66.81 ± 4.36	Medium
01GRAJ007I	Dominga	W/T	Green yellow	67.69 ± 28.24	Medium
01GRAJ013E	Maturana Blanca	W	Green yellow	68.37 ± 14.33	Medium
01GRAJ020I	Ondarrabi Beltza	W/T	Blue black	68.76 ± 19.75	Medium
01GRAJ019A	Jacquez	W	Blue black	69.80 ± 7.10	Medium
01GRAJ010H	Aligote	W	Green yellow	69.99 ± 3.98	Medium
01GRAJ011A	Cayetana Blanca	W/T	Green yellow	70.82 ± 0.54	Medium
01GRAJ022E	Schiava Grossa	W/T	Blue black	75.52 ± 1.19	High
01GRAJ020G	Negral	W	Blue black	76.48 ± 2.56	High
01GRAJ021J	Syrah	W	Blue black	77.33 ± 0.53	High
01GRAJ014L	Semillon	W	Green yellow	78.09 ± 1.44	High
01GRAJ017F	Beba Roja	W/T	Red	79.00 ± 2.46	High
01GRAJ017A	Alvarelhao	W	Blue black	80.51 ± 14.82	High
01GRAJ015B	Alarije	W	Green yellow	80.87 ± 7.65	High
01GRAJ009K	Naparo	T	Red	81.21 ± 16.76	High
01GRAJ020F	Icod de los Vinos	W	Blue black	81.39 ± 1.40	High
01GRAJ010C	Ruby Seedless	T	Rose	81.49 ± 1.29	High
01GRAJ015K	Verdil	W	Green yellow	82.70 ± 0.00	High
01GRAJ018A	Branco Escola	W	Blue black	82.71 ± 8.43	High
01GRAJ014E	Pedro Ximenes	W	Green yellow	82.88 ± 1.22	High
01GRAJ016D	Xarello	W	Green yellow	83.43 ± 1.44	High
01GRAJ019L	Listán Prieto	W	Blue black	83.53 ± 4.01	High
01GRAJ020C	Moravia Agria	W	Blue black	83.69 ± 1.25	High
01GRAJ008A	Pardillo	W	Green yellow	83.82 ± 1.13	High
01GRAJ007G	Afus Ali	W/T	Green yellow	84.74 ± 5.66	High
01GRAJ014A	Palomino de Jerez	W	Green yellow	85.37 ± 5.05	High
01GRAJ021D	Rubired	W/T	Blue black	85.59 ± 10.00	High
01GRAJ012K	Alcanon	W	Green yellow	86.00 ± 3.54	High
01GRAJ012F	Garrido Fino	W/T	Green yellow	86.30 ± 2.95	High
01GRAJ008D	Muscat Ottonel	W/T	Green yellow	86.95 ± 5.68	High
01GRAJ022A	Graciano	W	Blue black	87.07 ± 6.89	High
01GRAJ021I	Sumoll	W	Blue black	87.40 ± 7.99	High
01GRAJ011E	Chardonnay Blanc	W	Green yellow	87.55 ± 0.56	High
01GRAJ007C	Beba	W/T	Green yellow	87.65 ± 2.04	High
01GRAJ013G	Morio Muskat	W	Green yellow	87.83 ± 3.25	High
01GRAJ014G	Cornichon Blanc	W/T	Green yellow	88.10 ± 1.61	High
01GRAJ012B	Colombard	W/T	Green yellow	88.40 ± 3.35	High
01GRAJ018F	Gamay Noir	W	Blue black	88.43 ± 9.43	High
01GRAJ020B	Monastrell	W/T	Blue black	89.02 ± 0.68	High
01GRAJ008J	Quiebratinajas Blanco	T	Red	89.53 ± 10.68	High
01GRAJ011H	Siria	W	Green yellow	89.53 ± 2.90	High
01GRAJ017E	Valenci Tinto	W/T	Blue black	89.98 ± 1.43	High
01GRAJ013H	Muscat Hamburg	W/T	Green yellow	90.06 ± 0.05	Very high
01GRAJ015C	Silvaner Gruen	W	Green yellow	90.66 ± 1.88	Very high
01GRAJ018E	Tinto Velasco	W	Blue black	90.94 ± 2.12	Very high
01GRAJ019J	Mencía	W	Blue black	91.35 ± 0.63	Very high
01GRAJ007J	Mantuo	W/T	Green yellow	91.56 ± 10.21	Very high
01GRAJ016L	Trepat	W	Dark red violet	91.66 ± 0.62	Very high
01GRAJ012G	Gewuerztraminer	W	Green yellow	91.87 ± 5.06	Very high
01GRAJ020D	Aramon Noir	W/T	Blue black	92.02 ± 0.08	Very high
01GRAJ012L	Palomino Fino	W	Blue black	92.07 ± 2.88	Very high
01GRAJ009E	Cardinal	W/T	Red	92.45 ± 7.33	Very high
01GRAJ013B	Airen	W/T	Green yellow	92.66 ± 1.96	Very high
01GRAJ008I	Planta Nova	W/T	Green yellow	92.81 ± 4.52	Very high
01GRAJ022G	Valenci Tinto	W/T	Blue black	92.93 ± 0.42	Very high
01GRAJ009C	Alphonse Lavallee	W/T/R	Dark red violet	92.99 ± 1.03	Very high
01GRAJ016G	Alicante Henri Bouschet	W	Blue black	93.01 ± 5.48	Very high
01GRAJ021E	Ruby Cabernet	W/T	Blue black	93.07 ± 0.33	Very high
01GRAJ016F	Alfrocheiro	W	Blue black	93.38 ± 0.38	Very high
01GRAJ016J	Barbera Nera	W	Blue black	93.62 ± 1.87	Very high
01GRAJ007H	Delight	T	Green yellow	93.71 ± 2.45	Very high
01GRAJ017C	Cabernet Sauvignon	W	Blue black	94.04 ± 2.10	Very high
01GRAJ007L	Rey	W/T	Green yellow	94.25 ± 1.58	Very high
01GRAJ014I	Planta Fina	W/T	Green yellow	94.33 ± 0.96	Very high
01GRAJ007A	Aledo	T	Green yellow	94.55 ± 1.08	Very high
01GRAJ007F	Cornichón Blanco Falso	W/T	Green yellow	94.74 ± 1.65	Very high
01GRAJ019B	Moristel	W	Blue black	94.87 ± 1.28	Very high
01GRAJ014J	Riesling Weiss	W	Green yellow	95.13 ± 3.21	Very high
01GRAJ015G	Trajadura	W	Green yellow	95.25 ± 0.35	Very high
01GRAJ017G	Carnelian	W	Blue black	95.28 ± 0.77	Very high
01GRAJ013A	Loureiro Blanco	W	Green yellow	95.33 ± 0.83	Very high
01GRAJ018C	Fogoneau	W	Blue black	95.36 ± 1.81	Very high
01GRAJ020J	Graciano	W	Blue black	95.38 ± 0.88	Very high
01GRAJ019I	Mencía	W	Blue black	95.52 ± 0.63	Very high
01GRAJ011K	Verdejo Blanco	W	Green yellow	95.53 ± 2.31	Very high
01GRAJ012H	Siria	W	Green yellow	95.57 ± 1.57	Very high
01GRAJ009F	Cinsaut	W/T	Blue black	95.70 ± 0.25	Very high
01GRAJ011I	Clairette Blanche	W/T	Green yellow	95.71 ± 0.36	Very high
01GRAJ021H	Vinhao	W	Blue black	95.73 ± 0.10	Very high
01GRAJ017J	Centurion	W	Blue black	95.93 ± 0.68	Very high
01GRAJ011L	Folle Blanche	W	Green yellow	95.96 ± 2.62	Very high
01GRAJ018D	Forcallat Tinta	W	Blue black	96.12 ± 1.42	Very high
01GRAJ015E	Turruntés	W	Green yellow	96.21 ± 0.35	Very high
01GRAJ021B	Pinot	W	Blue black	96.48 ± 4.38	Very high
01GRAJ020L	Bouschet Petit	W	Blue black	96.72 ± 0.06	Very high
01GRAJ022F	Valdiguie	W	Blue black	96.82 ± 1.27	Very high
01GRAJ017D	Borracal	W	Blue black	96.84 ± 1.45	Very high
01GRAJ014K	Sauvignon Blanc	W	Green yellow	96.97 ± 2.05	Very high
01GRAJ020K	Castelao	W	Blue black	97.08 ± 0.34	Very high
01GRAJ016K	Bobal	W	Blue black	97.09 ± 0.03	Very high
01GRAJ010J	Auxerrois	W	Green yellow	97.23 ± 0.16	Very high
01GRAJ014F	Zalema	W	Green yellow	97.33 ± 0.46	Very high
01GRAJ010E	Alvarinho	W	Green yellow	97.50 ± 0.90	Very high
01GRAJ017B	Cabernet Franc	W	Blue black	97.74 ± 0.50	Very high
01GRAJ008F	Muskat Usbekistanskii	T	Green yellow	97.80 ± 1.37	Very high
01GRAJ016I	Aubun	W	Blue black	98.05 ± 1.87	Very high
01GRAJ021C	Cuelga	W	Green yellow	98.25 ± 0.54	Very high

For pollen viability, APV (%) mean ± SD (between inflorescences) are provided. Data from 2015

Main use: W, Wine grape; T, Table grape; R, Raisins [according to the *Vitis* International Variety Catalogue (*V*IVC, http://www.vivc.de)]. Grape skin color was evaluated following the Organisation Internationale de la Vigne et du Vin (OIV) descriptor N° 225. Pollen viability was rated as very high (APV > 90%), high (APV: 90–75%), medium (APV: 75–50%), low (APV: 50–25% and very low (APV < 25%)

**Fig. 7:**
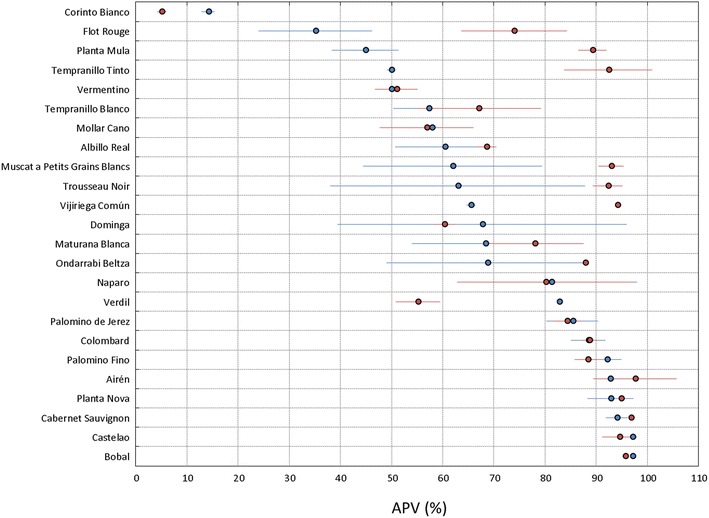
Automatic pollen viability rates [APV (%)] for 24 grapevine cultivars in 2015 (blue) and 2017 (red) obtained with PollenCounter. Dots indicate average values, whereas whiskers show standard deviations between inflorescences

Lastly, 24 cultivars were selected to phenotype this trait in a second season (2017). They included cultivars which in 2015 showed either low/very low pollen viability (like Corinto Bianco, Flot Rouge and Planta Mula) or high pollen viability difference between inflorescences (like Dominga, Maturana Blanca, Ondarrabi Beltza), as well as some randomly chosen cultivars with very high pollen viability (like Bobal, Castelao, Cabernet Sauvignon). On average, an increase in pollen viability (9.05%) was observed in this set of cultivars in 2017 compared to 2015, although important differences between cultivars were obtained (Fig. 7). They can be split into two groups according to their variation between seasons. One group includes 8 cultivars (Flot Rouge, Muscat a Petits Grains Blancs, Ondarrabi Beltza, Planta Mula, Tempranillo Tinto, Trousseau Noir, Verdil and Vijiriega Común) with an APV (%) difference between seasons larger than 10%. The rest of cultivars (Airén, Albillo Real, Bobal, Cabernet Sauvignon, Castelao, Colombard, Corinto Bianco, Dominga, Maturana Blanca, Mollar Cano, Naparo, Palomino de Jerez, Palomino Fino, Planta Nova, Tempranillo Blanco and Vermentino) show a lower difference between seasons. In the second group, we found diverse cultivars in terms of their APV (%) value, including very low: Corinto Bianco; low: Vermentino; medium: Albillo Real, Dominga, Mollar Cano and Tempranillo Blanco; high: Colombard, Naparo and Palomino de Jerez; and very high: Airén, Bobal, Cabernet Sauvignon, Castelao and Planta Nova.

## Discussion

### PollenCounter: a new tool for pollen viability phenotyping

Phenotyping is a critical component of plant genetic research and crop improvement. Accurate data collection, if combined with genetic information, can greatly accelerate progress in breeding for yield and quality traits of new and better adapted cultivars [23]. Recent works have reported the use of high-throughput technologies for the phenotyping of diverse grapevine traits, including plant phenology [36], crop yield components [36–38], grape quality [35, 39, 40] and fungal disease resistance [41–43]. In this context, the use of efficient and objective image-based systems for pollen viability estimation may provide an alternative solution for the time-consuming phenotyping of this trait. Noticing the variable size of viable and non-viable pollen grains in *M. guttatus* and *C. verna*, Kelly et al. [21] proposed the use of the pollen size index (PSI) as an indicator of pollen viability. In grapevine, we also observed a smaller size in non-viable pollen grains compared to viable ones, but the use of the PSI indicator yielded a non-significant correlation with the manual measurement of pollen viability (Additional file 4B), probably due to the substantial genetic variation in pollen grains size. As an example, the area of the smallest viable pollen grain ranged from 100 to 181 pixels^2^ (for cvs. Syrah and Vijiriega Común, respectively) in a subsample of 38 images from 19 cultivars. Although rather small, the detailed analysis of this subsample was enough to confirm that the establishment of a global surface area threshold for the automatic classification of pollen grains as viable or non-viable is not satisfactory enough to automate PSI calculation (and its subsequent pollen viability estimation) in grapevine wide genetic frameworks. Consequently, a genotype-specific evaluation of viable and non-viable pollen grains surface area distribution will be needed to set case-to-case size thresholds, hindering the automation of the process and its application to inter-genotypic studies.

In contrast to the great multi-cultivar variability detected for the pollen grain size, we observed that pollen grains looked similarly in all cultivars after Alexander’s staining. In all cultivars, viable and non-viable pollen grains were easily visually differentiated (dark blue = viable/pale blue = non-viable), suggesting the possibility of automating their differential counting through the use of novel image-based technologies on color digital microscope images. Any color digital image is the result of the combination of three primary color-channels [red (R), green (G) and blue (B)], which imitate the way humans perceive color [44]. These three channels store complementary information, and their analysis might yield different biological information for subsequent decision support. In our case, R, G and B channels contain differential information on viable and non-viable pollen grains (which correspond to the differential R, G and B profiles of the stains included in Alexander’s modified staining) which can be used for its separation and further independent analysis. In this regard, the separation algorithm of the RGB images into independent colors channels is a critical step towards automatic image techniques, and so different separation processes have been proposed [44]. Here, we used the separation of the red, green and blue primary channels of the original RGB color digital microscope images to represent the intensity of the stains. Although color separation was not perfect (the color spectrum of both malachite green and acid fuchsin dyes spread over other color channels), we obtained enough selective contrast between these two dyes in the red and green layers to provide an appropriate difference between light blue- and dark purple-stained structures. In this regard, diverse processes (like stain deconvolution) have been proposed as alternative approaches to improve the separation of stains [45], but prior information is needed about the red, green and blue components of the pure stains to separate, which is not always feasible [46]. The simple separation approach used in our work and implemented in PollenCounter proved to be sufficient to make an appropriate counting of viable and total pollen grains in a diverse genetic framework, yielding highly accurate viability measurements and replacing labor-intensive and time-demanding manual processes.

PollenCounter can be easily run by users by opening the macro (Additional file 2) with Fiji and by choosing the source directory where the pollen digital images are stored. Then, the macro automatically analyses the whole set of images, releasing the number of pollen grains counted in the red and green color fractionated images in a pivot table for pollen viability calculation. Obviously, an adequate contrast between viable and non-viable pollen grains is needed when acquiring the RGB images, and settings should be optimized by the user to obtain the best differentiation of both type of pollen grains. The macro can be adapted to user needs by modifying different parameters related to pollen grain morphology, like their size and circularity. In fact, this flexibility opens its use to the evaluation of pollen viability in other crops with different pollen grain size and shape. As default settings, we set a surface area range of 60–800 pixels^2^ for the total counting of grapevine pollen grains (either viable or non-viable) in the red channel. In contrast, and aware of the general higher size of viable grains compared to non-viable, this range was slightly shortened to 100–800 pixels^2^ for the counting of viable pollen grains in the green channel, since no viable pollen grains were generally observed under 100 pixels^2^ and some small dark-stained particles (other than pollen grains) can appear as the result of the staining procedure. Thus, the presence of an adjacent small acid fuchsin-stained balloon-like structure next to some viable pollen grains was found to be common in some cultivars like Doña Blanca, Muscat Ottonel or Tinto Velasco (Additional file 7A). This structure could correspond to some protoplasm content released after intine rupture due to pollen grain hydration during the staining procedure, as previously suggested [47]. Although these structures are discarded during the processing of red-channel derived images due to their color features (Additional files 7B and 7D), they are detected in the green channel (Additional file 7C), so potentially counted as viable pollen grains if no area filter is included. The area filter included in our tool allows discarding these particles (Additional file 7E), decreasing the number of false positives and improving the accuracy of our tool.

Additionally, circularity factor can be modified in PollenCounter to adapt to pollen shape features. In general, we observed that non-viable pollen grains tend to be less spherical than viable grains (see Additional file 5 for an example). Nonetheless, such difference was not wide enough to establish it as an additional criterion for pollen grains differentiation, so we opted to maintain a fixed wide range of 0.40–1.00 to ensure a wide-spectrum counting of pollen grains.

### Pollen viability shows a high uniformity between different inflorescences of the same clone

Nowadays, certified grapevine clones are asexually multiplied in nurseries by vegetative propagation to obtain genetically identical copies. During this process, the frequency and rate of spontaneous mutations driving to divergent phenotypes are suggested to be low [48], and so the vast majority of the new plant material obtained shows identical phenotypic features to the original plant. Accordingly, we observed similar pollen viability values for the different plants sampled for Cabernet Franc, Chardonnay, Gewuerztraminer and Sauvignon Blanc genotypes, which respectively derived from a single clone. Besides, diverse works indicate that shoot and inflorescence position can influence grapevine reproductive performance. As some examples, basal clusters on Vidal blanc and Concord hybrids have been reported to be significantly heavier than second order clusters [49, 50], and similar differences have been found in Cabernet Sauvignon, Merlot and Sauvignon blanc *V. vinifera* cultivars [51, 52]. Phenology is another trait suggested to be affected by cluster and shoot positions, with lower inflorescences emerging before those in an upper position, and distal shoots flowering before proximal ones [52, 53]. Here, we aimed to evaluate the contribution of shoot and inflorescence position to pollen viability variability in four different genetic backgrounds. Our results indicate that pollen viability values of Cabernet Franc, Chardonnay, Gewuerztraminer and Sauvignon Blanc are stable between different inflorescences, and we did not find any evidence of differences between basal and second clusters, nor between clusters from proximal and distal shoots. It is generally accepted that carbohydrate reserves are needed for pollen grain formation and development [13, 54], which could be compromised in some flowers due to the known preferential sugar transport to specific inflorescences during flowering time [52]. Unfortunately, carbohydrate reserves were not evaluated in our work, so it was not possible to establish a correlation between sugar level and pollen viability in inflorescences of different order/position. Anyway, it could be expected that carbohydrate reserves and/or supply during pollen formation were sufficient to ensure an adequate pollen development in these cases.

To our knowledge, the current work is the first one aimed to determine sources of variability within-plant (cluster-to-cluster differences on a shoot and shoot-to-shoot differences on a vine) in grapevine pollen viability through its direct quantification. The observed uniformity of pollen viability in the different inflorescences along the shoot and the cordon has relevant implications in sampling strategies. Our findings suggest that under optimal growing conditions, a random sampling of flowers from inflorescences from selected shoots is enough to obtain unbiased results to explore the genetic variability in pollen viability in diverse grapevine genotypes.

### Tempranillo Tinto clones show high pollen viability variability

Somatic variation can occasionally affect major phenotypic traits, and somatic variants can be exploited for clonal selection programs for the improvement of elite cultivars [48]. As seen for other traits like grape aroma [55], berry skin color [56], cluster compactness [57] or water use efficiency [58], we observed a wide intra-cultivar genetic variability for pollen viability. In this regard, clones of Tempranillo Tinto with very high (RJ-51 and VP-2) and much lower (VP-11 and VP-25) pollen viability values were identified within the same plot (VP). These four Tempranillo Tinto clones were intentionally selected for their different viticulture performances [33]: whereas RJ-51 and VP-2 are two high-yielding clones with compact clusters and a high number of berries per cluster, VP-11 and VP-25 are two less productive and less compact clones with a minor number of berries per cluster. As previously suggested for a set of Cabernet Sauvignon clones [8], our results indicate a direct link between pollen viability and crop yield. In this regard, high pollen sterility (and the probably linked ovule sterility [15]) may lower fruit set rates and berry number per cluster, reducing cluster weight and consequently, crop yield [1, 5]. Similarly, the reduction of the berry number will reduce cluster compactness, which in turn might improve grape and wine quality via cluster microclimate improvement [59].

Specific comparative cytological and transcriptome analyses between sterile mutants and fertile wild-types have been useful to uncover the role of different genetic networks in the pollen development of the model plant *A. thaliana* [60, 61]. In grapevine, such analyses are hindered by the lack of mutant collections [62]. An alternative approach is the comparative analysis of clones differing in the trait of interest, like recently done for the in-depth analysis of cluster compactness [57]. In this regard, the identification and further characterization of the Tempranillo Tinto clones described here may be very useful to understand the genetic mechanisms involved in male sterility determination in grapevine.

### Grapevine cultivars show large variability for pollen viability and different susceptibility to environment

The assessment of genetic diversity by exploring natural variation is the basis for the improvement of any crop. Over the last years, an increasing number of studies have dealt with the evaluation of grapevine diversity for many relevant traits, such as berry size [63], cluster structure [33] and water use efficiency [58]. The quality of the pollen produced by grapevine flowers is an important component of its reproductive behavior [5], but the information available is limited to a low number of genotypes [5, 7, 18, 64–66]. In this regard, our work aimed to explore the genetic diversity of this trait among a wide set of table- and wine-grape cultivars of diverse origin, showing for the first time the wide variability present in the cultivated grapevine.

In contrast to the uniformity observed for the pollen viability between plants in Cabernet Franc, Chardonnay, Gewuerztraminer and Sauvignon Blanc, we observed some cultivars with a high level of variation between the two plants (inflorescences) sampled in the ICVV plot. These plants were generated in 2009 from the duplication of an older grapevine collection grown in another plot [33]. During this process, different plants of the same cultivar were used to obtain the scions, which could explain part of the differences observed. Nevertheless, other additional genetic factors reducing pollen viability uniformity between inflorescences cannot be discarded.

As expected, most of the cultivars analyzed in this work showed high or very high pollen viability values, including very relevant wine grape varieties like Airén, Cabernet Sauvignon and Pinot Noir. During grapevine domestication and selection, humans focused on maintaining those genotypes capable to yield regular grape production, and so they unconsciously selected beneficial alleles for fertility, flower drop and productivity [67]. In this regard, self-fruitful hermaphroditic plants were preferentially selected over the dioecious wild form, since they allowed the obtaining of much more fruit on a predictable basis [68]. During this selection process, it is likely that grapevine genotypes with low pollen viability could have been progressively unwittingly discarded, maintaining those with better reproductive performance. Exceptionally, individuals with a low capability to generate viable pollen grains could have been selected for grape production despite this disadvantage. This can be the case of Corinto Bianco, a somatic variant of the seeded cultivar Pedro Ximenes [69] with very low pollen viability, but able to produce seedless berries without fertilization, which are highly appreciated for dried fruit consumption (raisins). Recently, an array of causes has been described to understand the genetic origin of parthenocarpy of Corinto Bianco [18], which include the absence of viable pollen. In contrast, we observed a low presence of viable pollen grains, which would explain the occasional formation of seeded berries after fertilization [18, 69]. Genetic and ploidy analysis of regenerated plantlets from Corinto Bianco seeds revealed their polyploidy (3n or 4n), probably derived by the fusion of unreduced gametes [18]. Interestingly, we observed an abnormal higher size for the viable pollen grains of this cultivar compared to non-viable pollen grains, especially in 2015 (Additional file 8). Considering the generally accepted correlation between pollen grain size and ploidy level [70], these stained and bigger pollen grains may correspond to viable diploid (2n) pollen grains of Corinto Bianco.

Pollen development and maturation is known to be sensitive to environmental conditions, and both abiotic (heat, cold, drought) and biotic factors may affect male reproductive performance [54]. In our work, 2015 and 2017 environmental conditions might have played specific effects in the different grapevine cultivars analyzed, identifying more and less-sensitive cultivars. Regarding the sensitive ones (those with a difference in APV (%) between seasons above 10%), we found higher pollen viability in 2017 than in 2015, except for the Spanish cultivar Verdil. Pre-flowering climate conditions were slightly different in La Rioja during these two seasons (2015 was warmer and drier than 2017, Additional file 9) and it could have been one of the factors affecting pollen development, as generally accepted for other crops [54, 71, 72]. In this light, previous reports indicate that the effect of temperature on pollen viability is genotype-specific [9, 27, 73], and very high temperatures (42 °C for 4 h) can jeopardize the regular formation of the outer wall of pollen grains in cv. Touriga Nacional, driving to an overall reduced viability [27]. Nevertheless, specific works are needed to understand how environmental factors individually affect grapevine pollen formation and/or maturation. Besides, and although many factors are involved, the sensitivity of these cultivars to temperature could explain part of the seasonal variance observed for some yield components for these genotypes, like previously reported for cv. Trousseau Noir [74].

## Conclusions

Pollen viability assessment is critical for understanding grapevine reproductive development and for grapevine genetic improvement. Here, we introduce PollenCounter, a new processing tool capable to do an automatic counting of pollen grains after Alexander’s modified staining. Its high precision and accuracy make it suitable to estimate pollen viability in a diverse genetic framework, substituting time-demanding manual processes. The automatic analysis of pollen viability in a wide collection of cultivars revealed a great genetic variability for this trait, as well as differences in their susceptibility to the environment. A large variation was also identified among Tempranillo Tinto clones, but very small within single plants of the same clone of diverse cultivars. Some of the insights given in this work will guide future analyses aimed to unravel the physiological and genetic mechanisms affecting pollen viability in grapevine.

## Additional files

**Additional file 1.** List of the 120 grapevine accessions selected for this work. Accession code, cultivar name, and season sampled are indicated.

**Additional file 2.** PollenCounter Fiji-compatible macro.

**Additional file 3.** Pollen images.

**Additional file 4.** Analysis of two different automatic methods to estimate pollen viability. In A, histograms are shown for non-viable (NV, blue) and viable (V, green) pollen grains area (pixels^2^). Pollen grains (n = 6082) contained in 38 images from 19 different cultivars are considered. Red broken line indicates the average value of the smallest viable pollen grain detected in each image (118 pixels^2^). In B, pollen size index (PSI_img_) is compared to the manual pollen viability (MPV_img_) assessment. In B, the manual approach is compared to the automatic system designed in this work (APV_img_). For B and C, 38 images are considered.

**Additional file 5.** Separation of the red (R), green (G) and blue (B) layers of a RGB image of six grapevine pollen grains (three viable, three non-viable). To obtain red, green and blue color fractionated grayscale pictures, the original RGB image was processed using the “split channels” tool in Fiji. Pollen grains correspond to cv. Chardonnay.

**Additional file 6.** Separation of RGB images of the three dyes used in the Alexander’s modified staining solution (AF: Acid fuchsin; MG: Malachite green; OG: Orange G) on their red (R), green (G) and blue (B) basic layers. To obtain R, G and B color fractionated grayscale pictures, RGB images of pure dyes were processed using the “split channels” tool in Fiji.

**Additional file 7.** Original (A), red- and green-color fractionated grayscale pictures (B, C), and PollenCounter output images (D, E) obtained for a pollen sample of cv. Muscat Ottonel. An example of protoplasm content released from a viable pollen grain is indicated with a red arrow. Note that it is not detected in the red-channel derived images (B, D). Although detected in the green channel (C), the structure is not considered as a valid pollen grain to be counted (black region, E). For simplification, only a representative area of the processed image is shown. B and C images were obtained using the “split channels” tool in Fiji. The number of pollen grains automatically counted by PollenCounter (in cyan) is indicated in the lower right corners (D, E).

**Additional file 8.** Corinto Bianco pollen grains after Alexander’s modified staining obtained in 2015 (A) and 2017 (B). Red arrows indicate viable pollen grains. For simplification, only representative areas are shown.

**Additional file 9.** Pre- and post-flowering climate conditions in the Grapevine Germplasm Collection of the Instituto de Ciencias de la Vid y del Vino in 2015 and 2017. Days are shown according to the date when the first sample was collected in 2015 (2-June) and 2017 (23-May), indicated as a “0”. Sample collection extended for 14 and 10 days in 2015 and 2017, respectively. Light and dark red lines indicate mean temperatures for 2015 and 2017, respectively. Light and dark blue columns indicate accumulate daily rainfalls for 2015 and 2017, respectively. Data were obtained from La Rioja Government website (http://www.larioja.org/siar).
