# Supplementary figures and images for: A new image-based tool for the high throughput phenotyping of pollen viability: evaluation of inter- and intra-cultivar diversity in grapevine
Source: Plant Methods. 2018 Jan 9;14:3. doi: 10.1186/s13007-017-0267-2 (PMC5759351; doi:10.1186/s13007-017-0267-2)

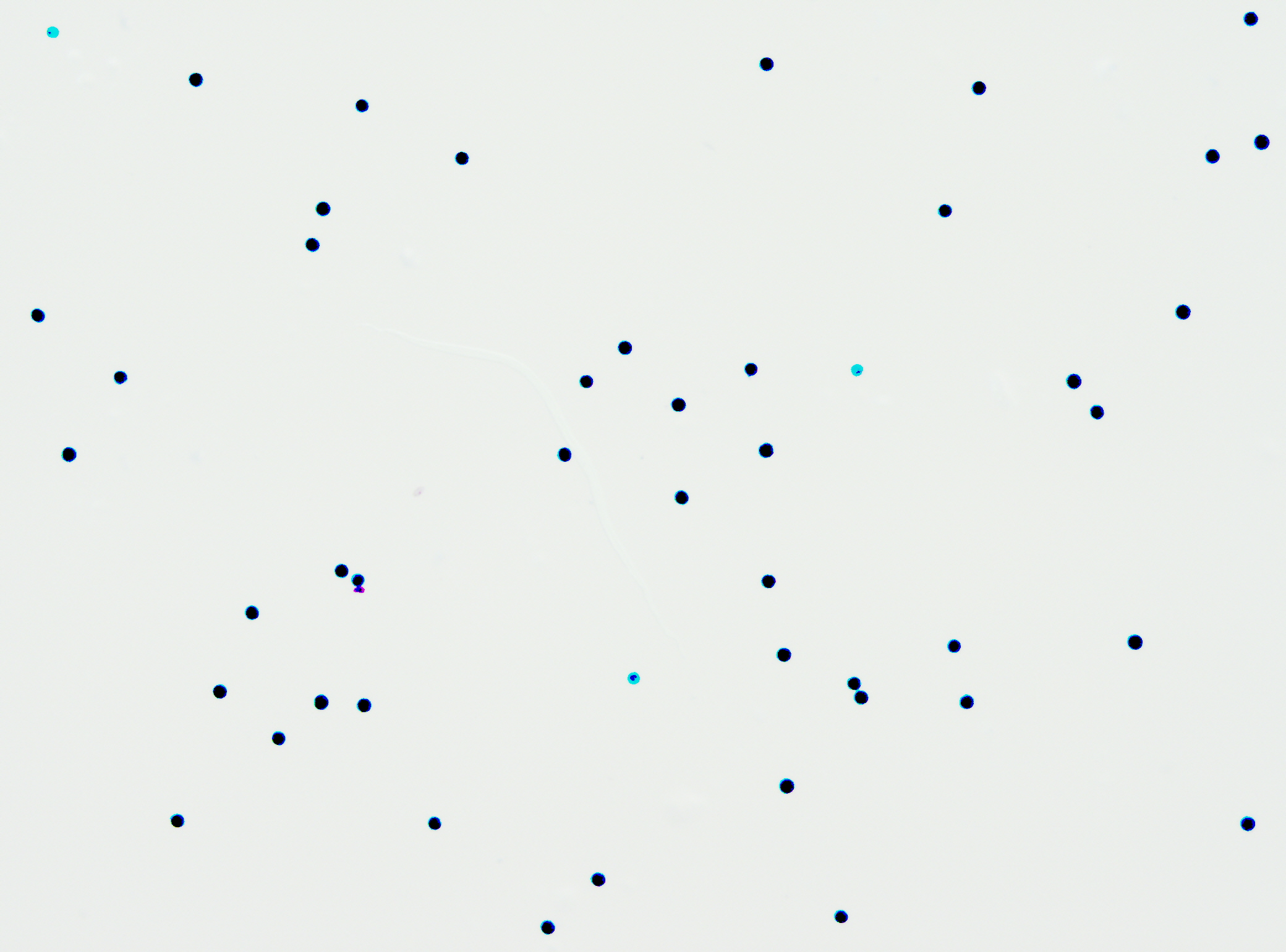

Supplement: Supplementary file 3 — Additional file 3. Pollen images. [file 13007_2017_267_MOESM3_ESM.zip › AddFile3/Vv_Pollen_Alcanon.JPG]

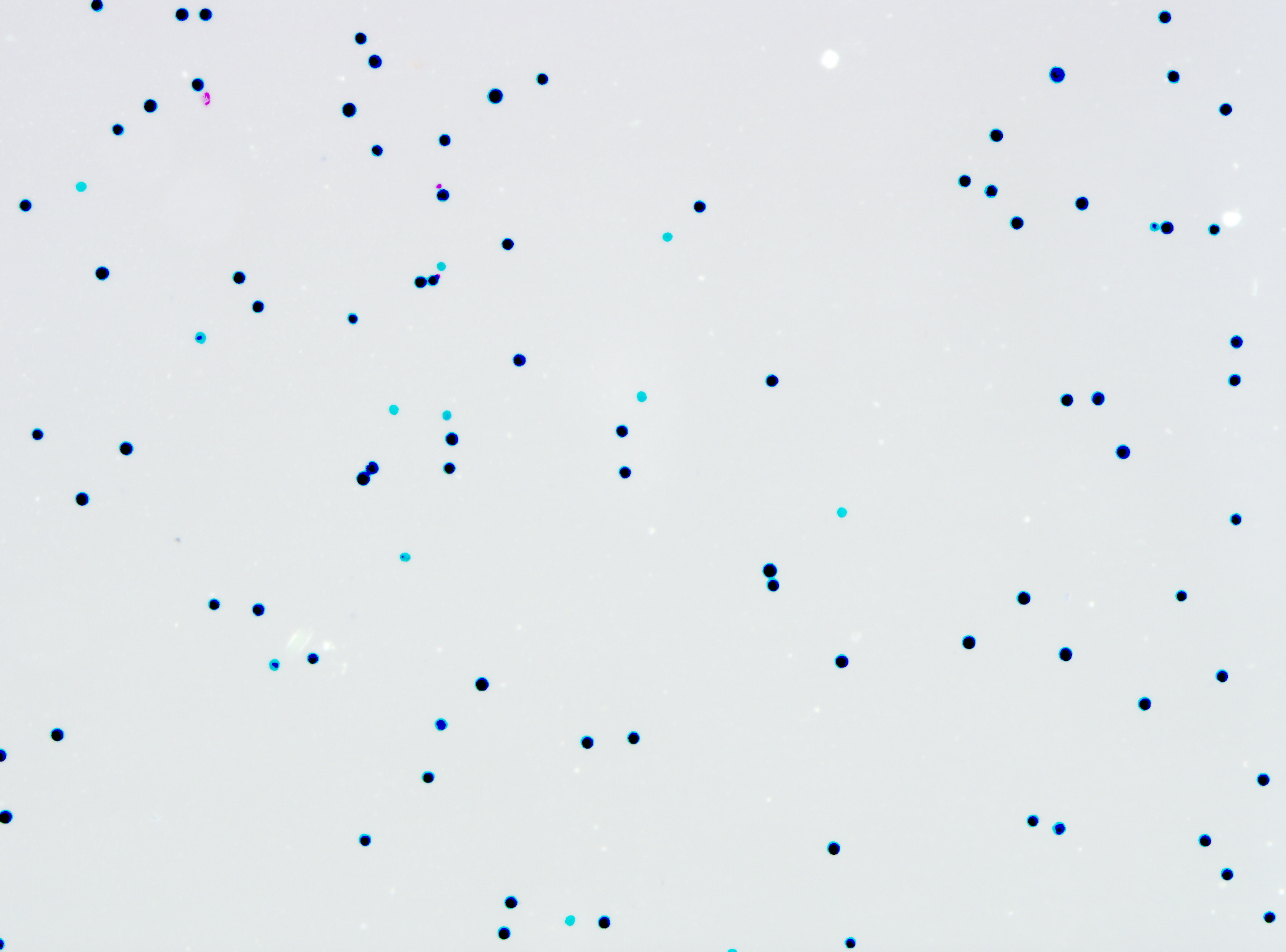

Supplement: Supplementary file 3 — Additional file 3. Pollen images. [file 13007_2017_267_MOESM3_ESM.zip › AddFile3/Vv_Pollen_Aligote.JPG]

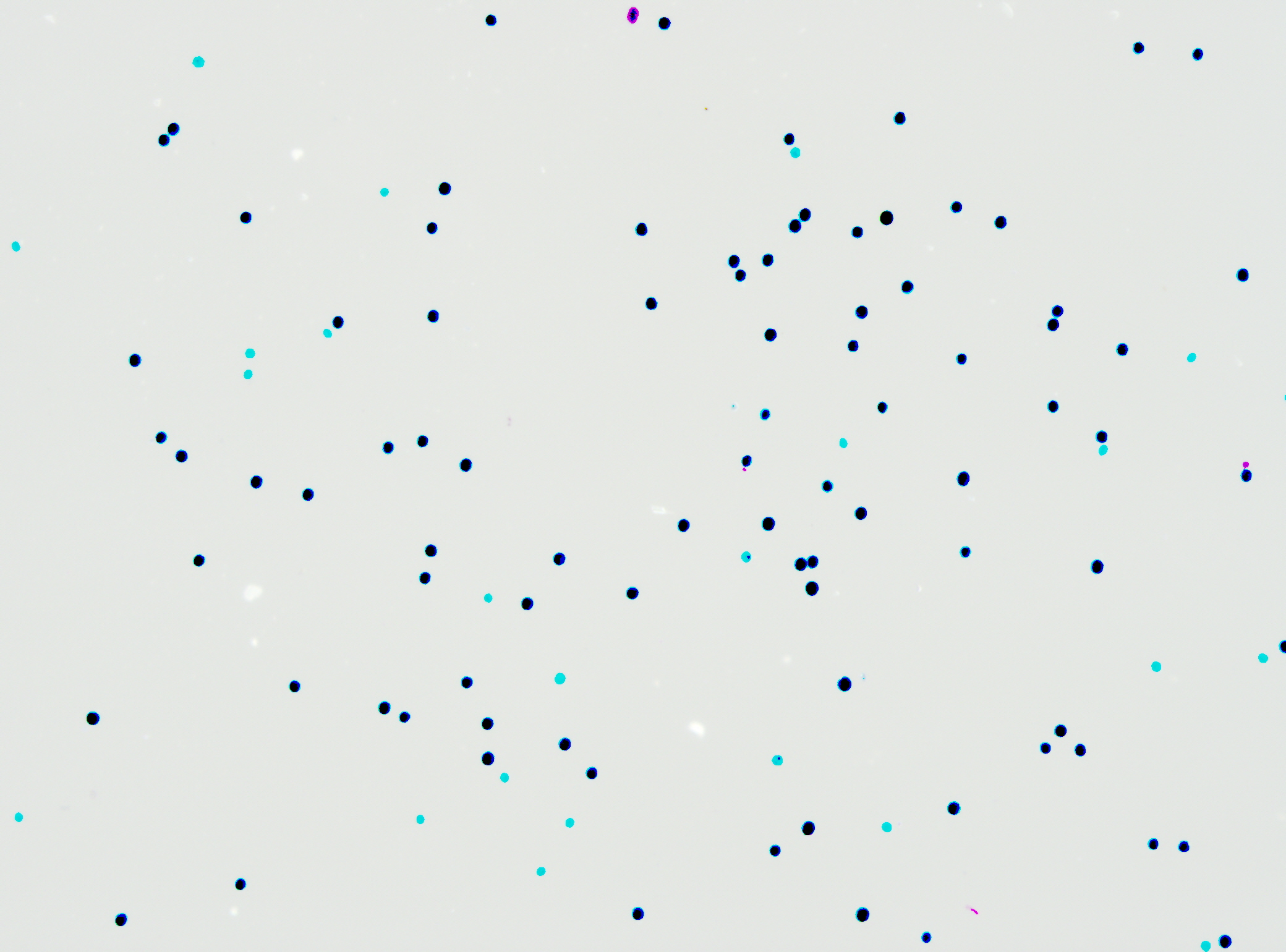

Supplement: Supplementary file 3 — Additional file 3. Pollen images. [file 13007_2017_267_MOESM3_ESM.zip › AddFile3/Vv_Pollen_Beba.JPG]

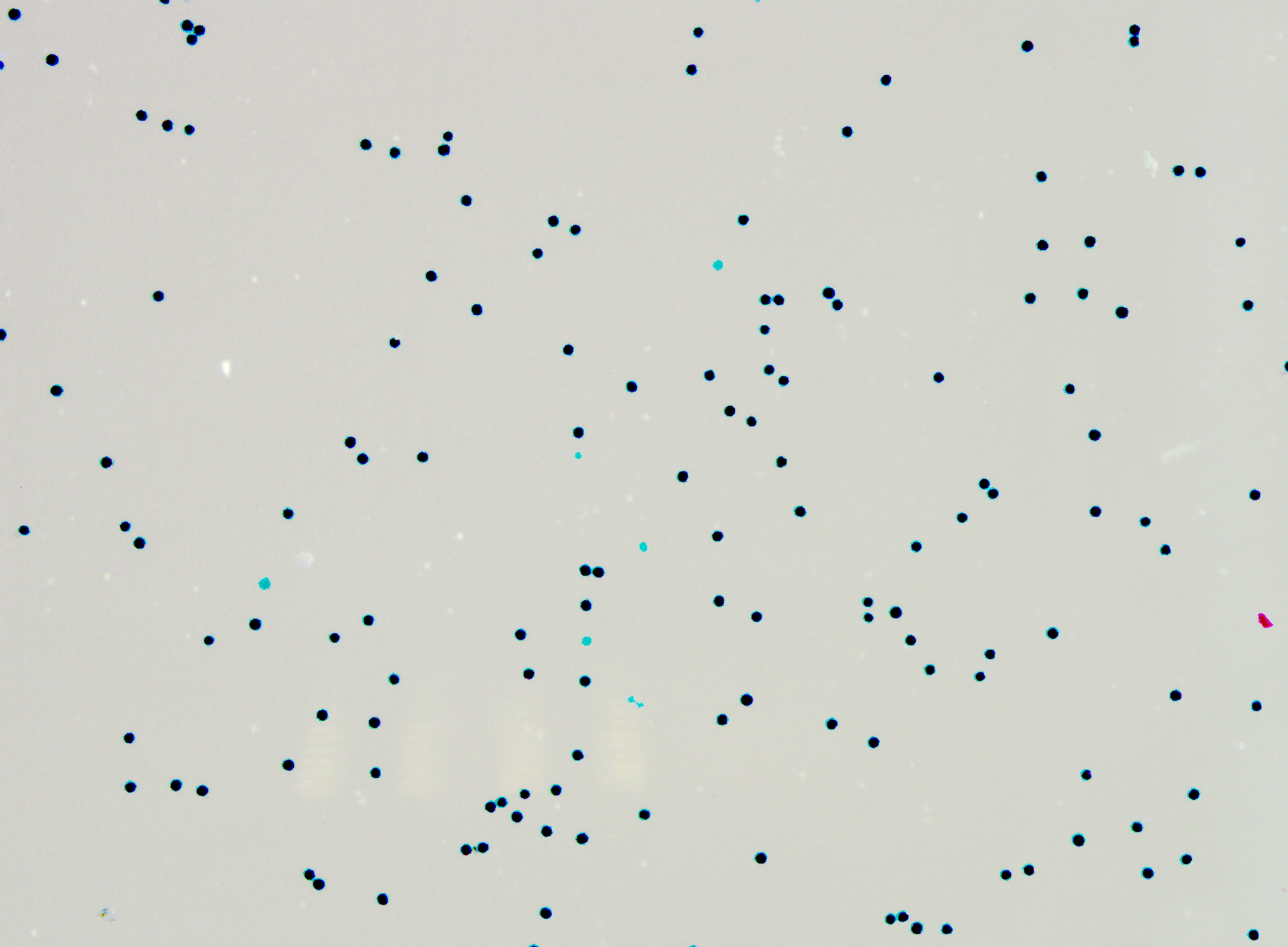

Supplement: Supplementary file 3 — Additional file 3. Pollen images. [file 13007_2017_267_MOESM3_ESM.zip › AddFile3/Vv_Pollen_Cabernet_Franc.JPG]

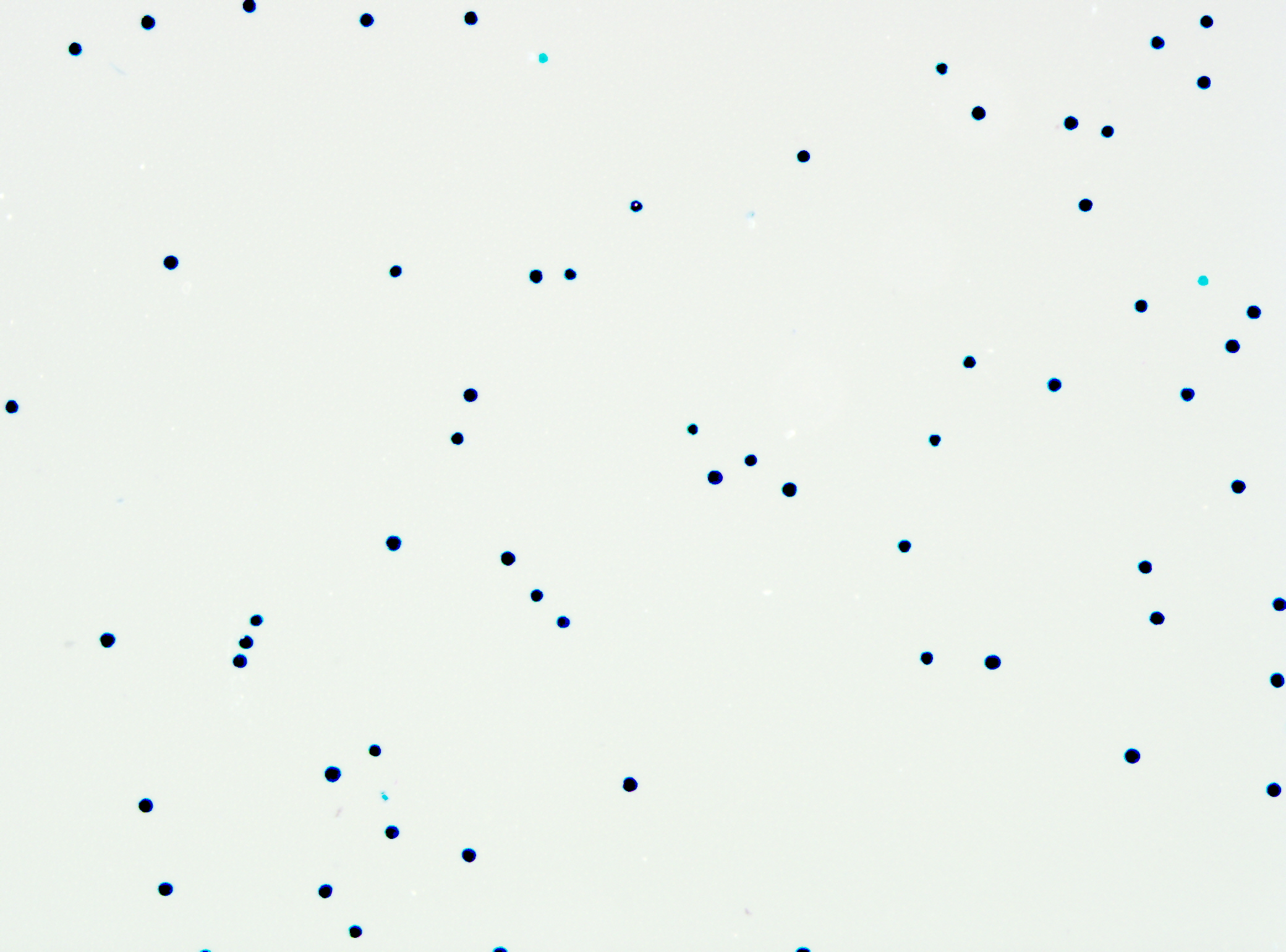

Supplement: Supplementary file 3 — Additional file 3. Pollen images. [file 13007_2017_267_MOESM3_ESM.zip › AddFile3/Vv_Pollen_Clairette_Blanche.JPG]

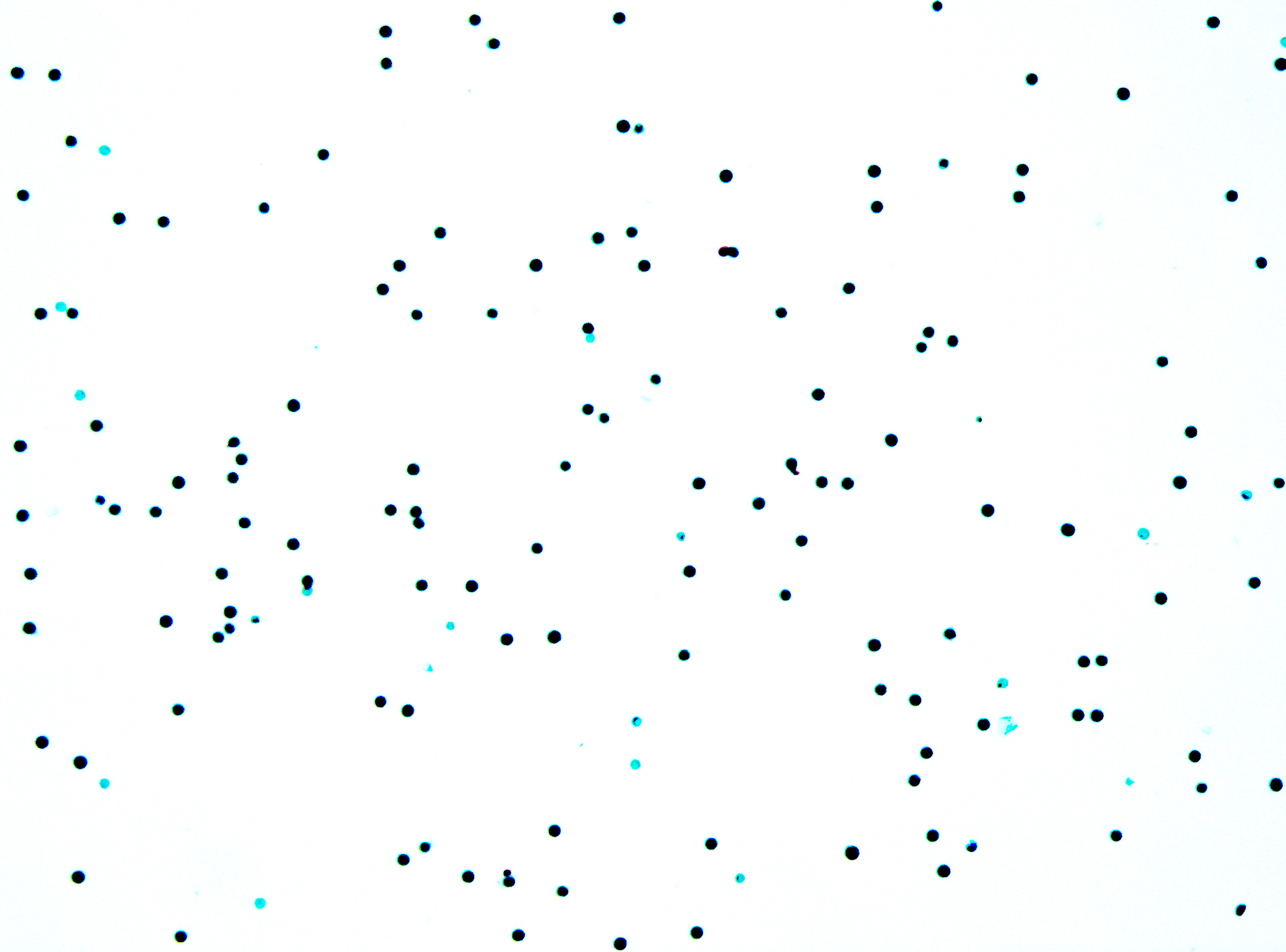

Supplement: Supplementary file 3 — Additional file 3. Pollen images. [file 13007_2017_267_MOESM3_ESM.zip › AddFile3/Vv_Pollen_Muscat_Hamburg.JPG]

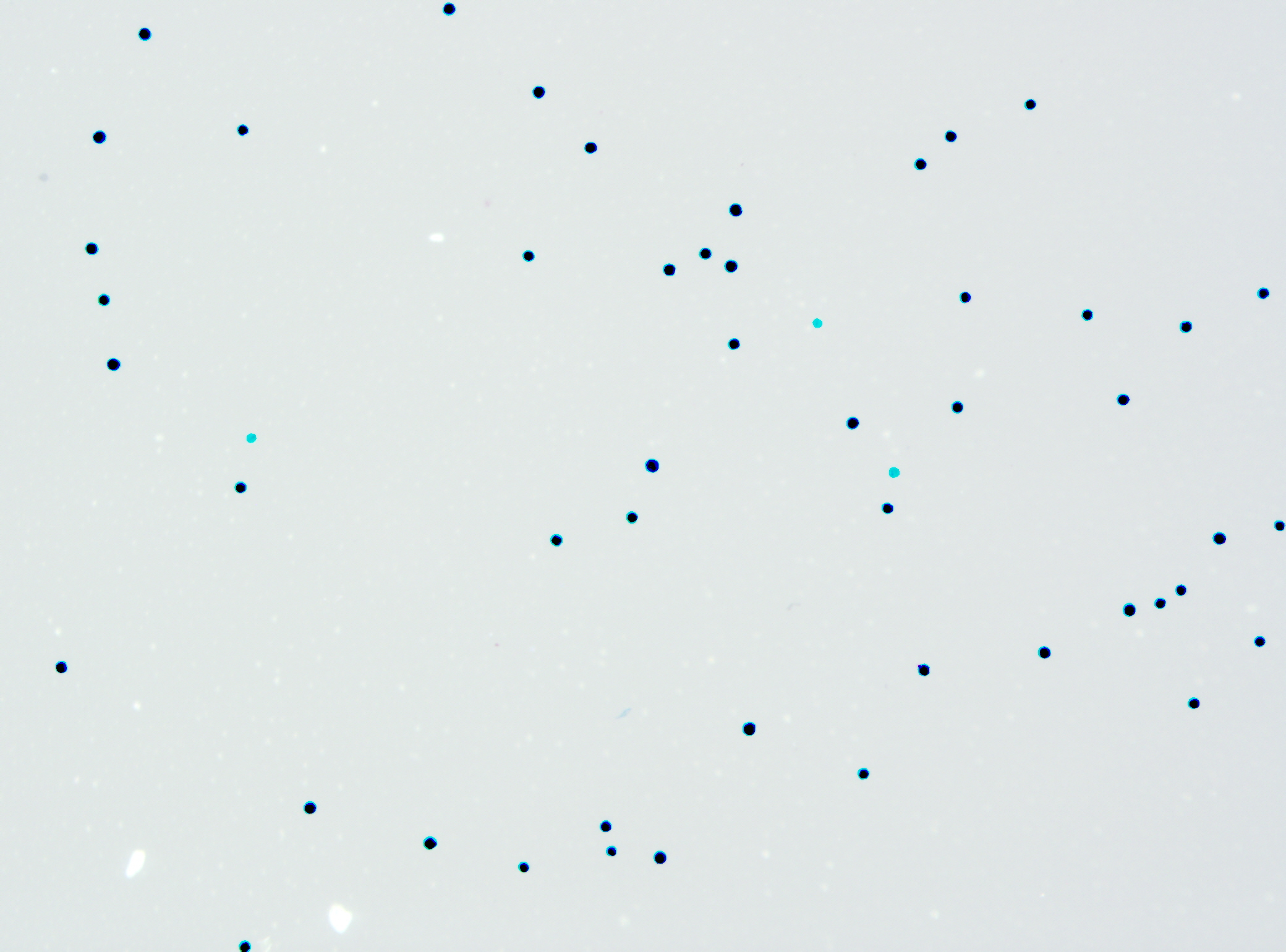

Supplement: Supplementary file 3 — Additional file 3. Pollen images. [file 13007_2017_267_MOESM3_ESM.zip › AddFile3/Vv_Pollen_Pedro_Ximenes.JPG]

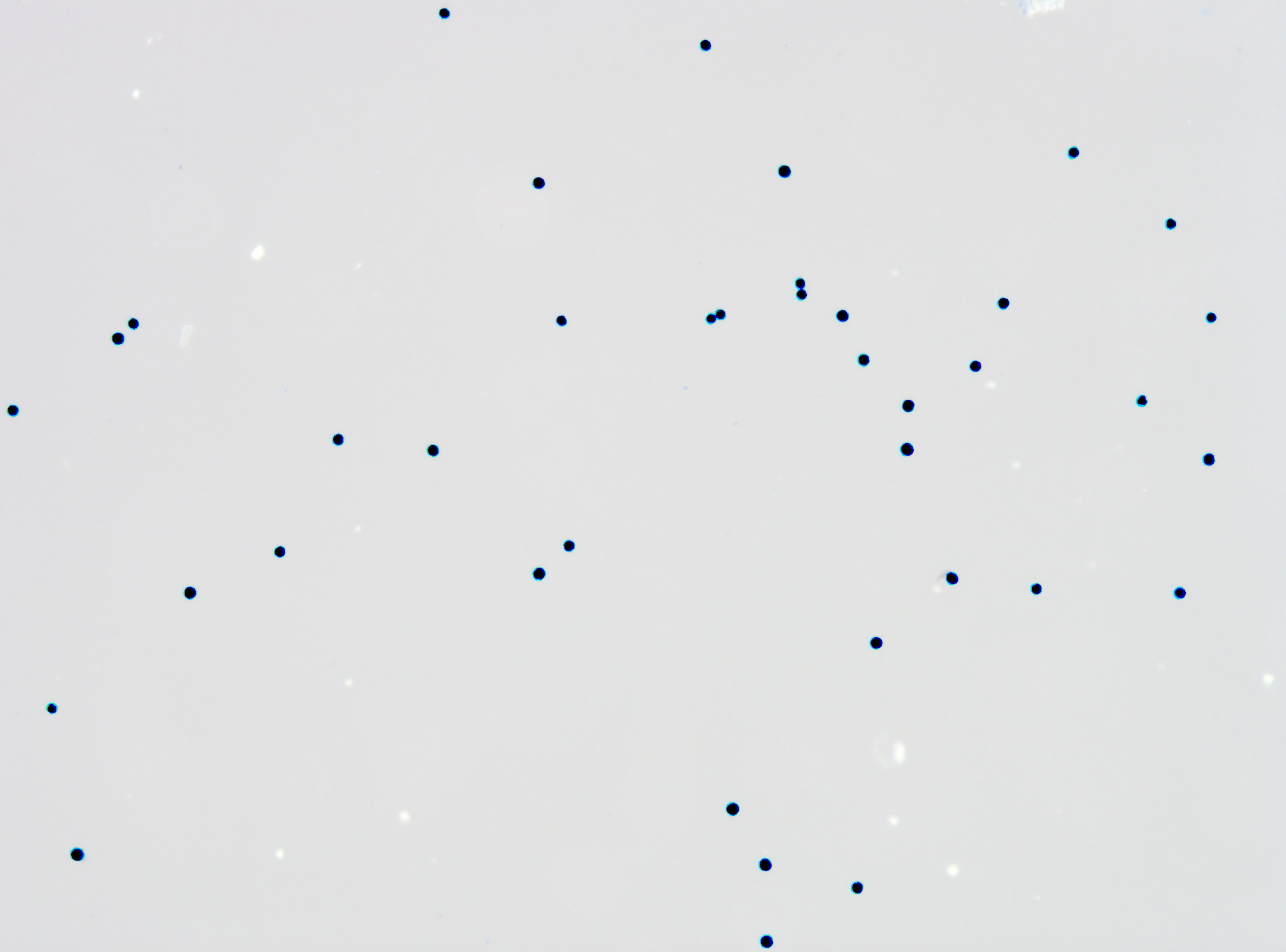

Supplement: Supplementary file 3 — Additional file 3. Pollen images. [file 13007_2017_267_MOESM3_ESM.zip › AddFile3/Vv_Pollen_Reisling_Weiss.JPG]

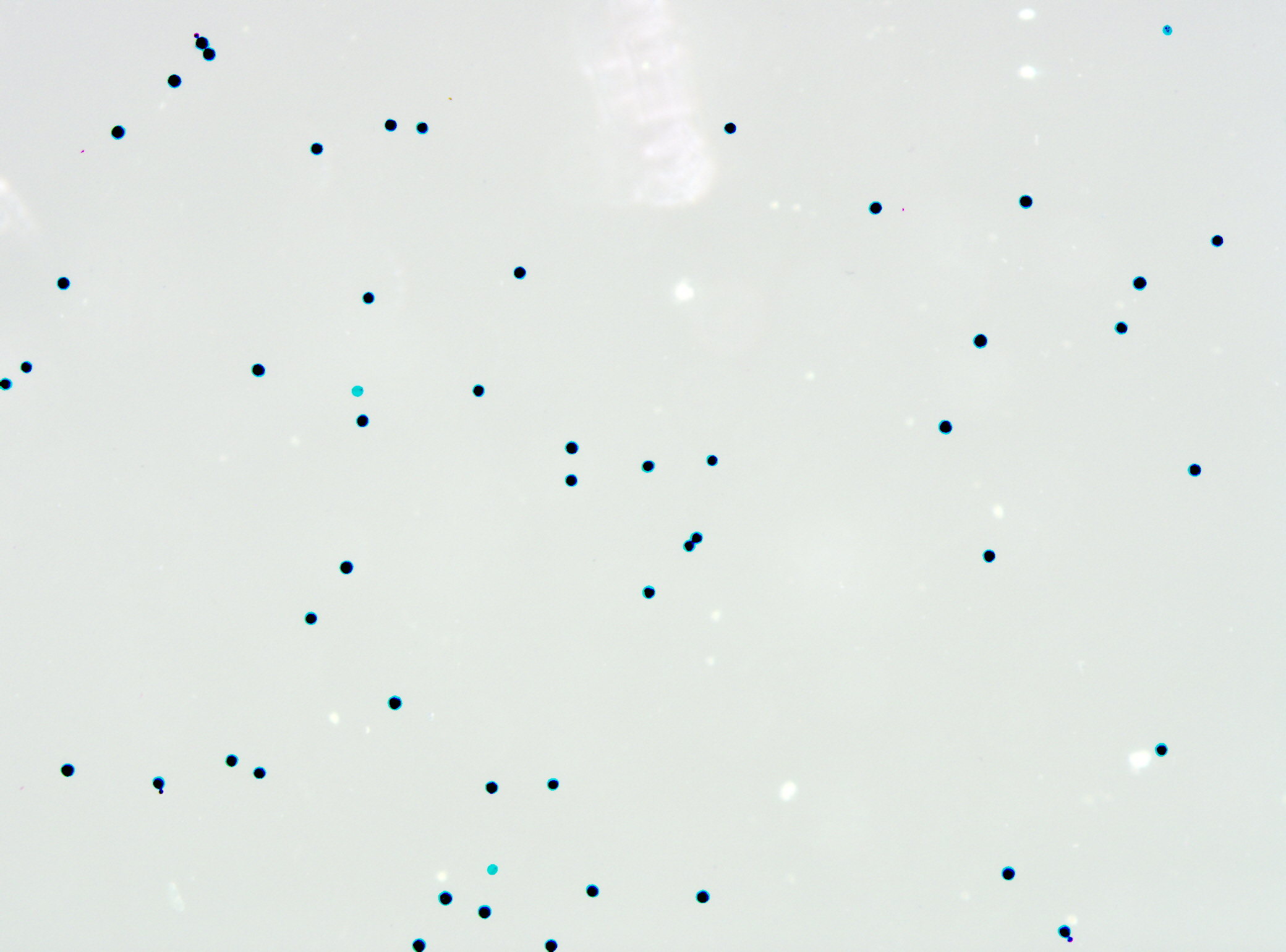

Supplement: Supplementary file 3 — Additional file 3. Pollen images. [file 13007_2017_267_MOESM3_ESM.zip › AddFile3/Vv_Pollen_Silvaner_Gruen.JPG]

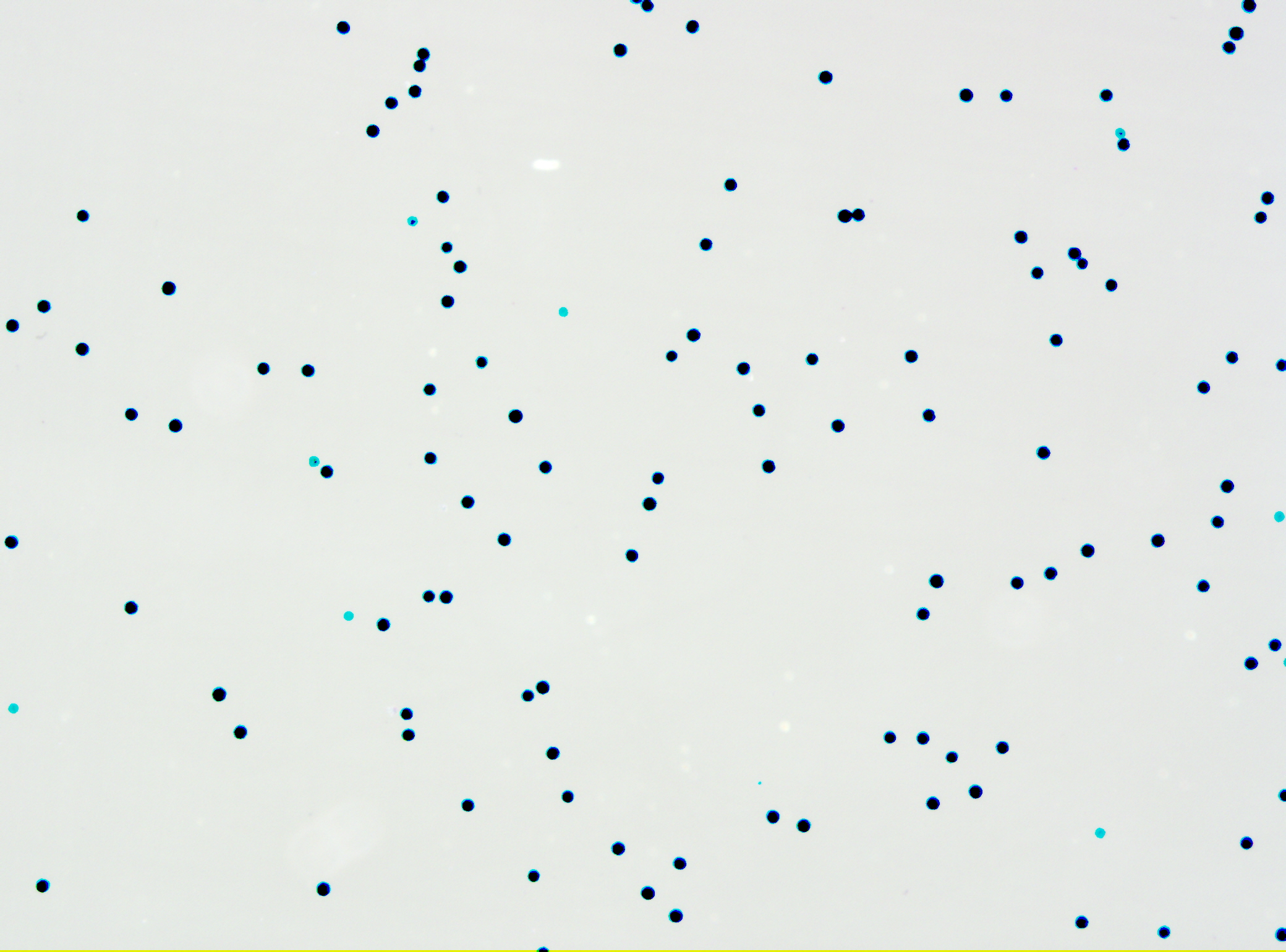

Supplement: Supplementary file 3 — Additional file 3. Pollen images. [file 13007_2017_267_MOESM3_ESM.zip › AddFile3/Vv_Pollen_Vinhao.JPG]

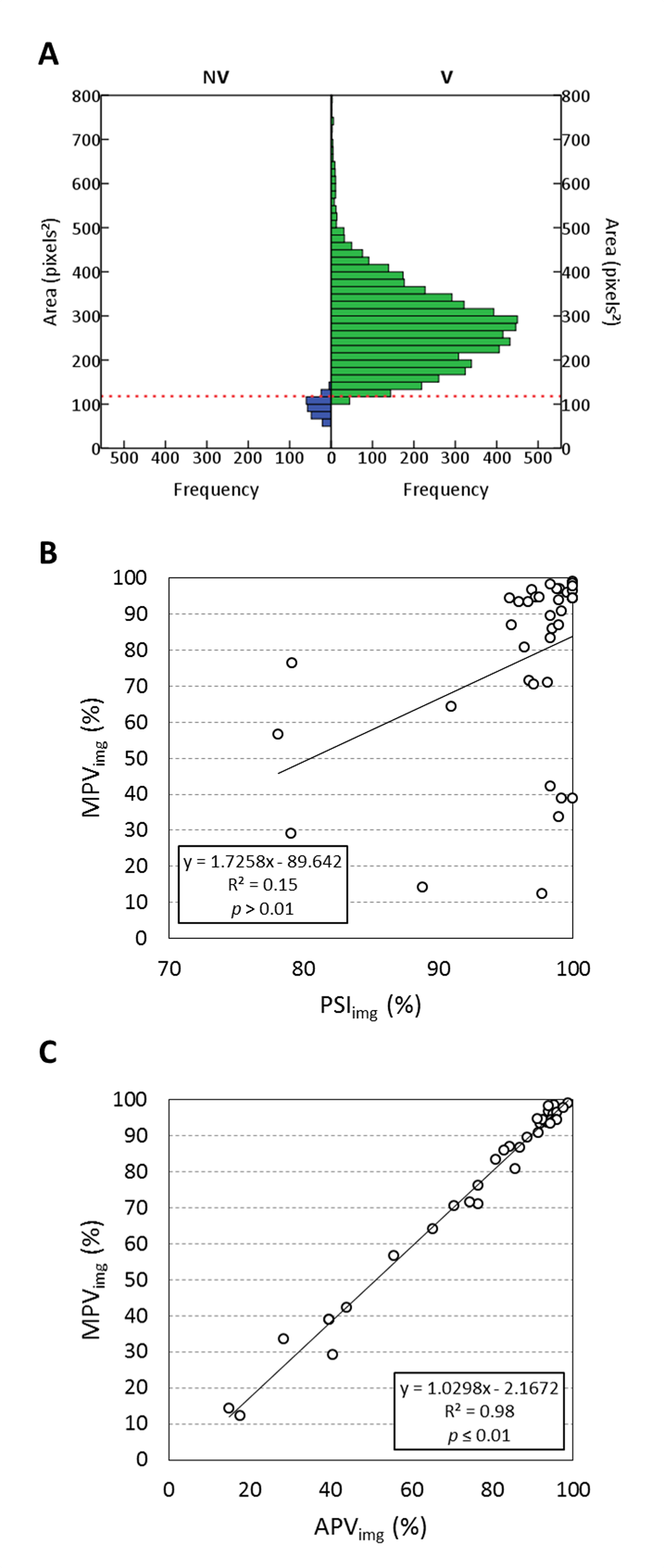

Supplement: Supplementary file 4 — Additional file 4. Analysis of two different automatic methods to estimate pollen viability. In A, histograms are shown for non-viable (NV, blue) and viable (V, green) pollen grains area (pixels2). Pollen grains (n = 6082) contained in 38 images from 19 different cultivars are considered. Red broken line indicates the average value of the smallest viable pollen grain detected in each image (118 pixels2). In B, pollen size index (PSIimg) is compared to the manual pollen viability (MPVimg) assessment. In B, the manual approach is compared to the automatic system designed in this work (APVimg). For B and C, 38 images are considered. [file 13007_2017_267_MOESM4_ESM.tif]

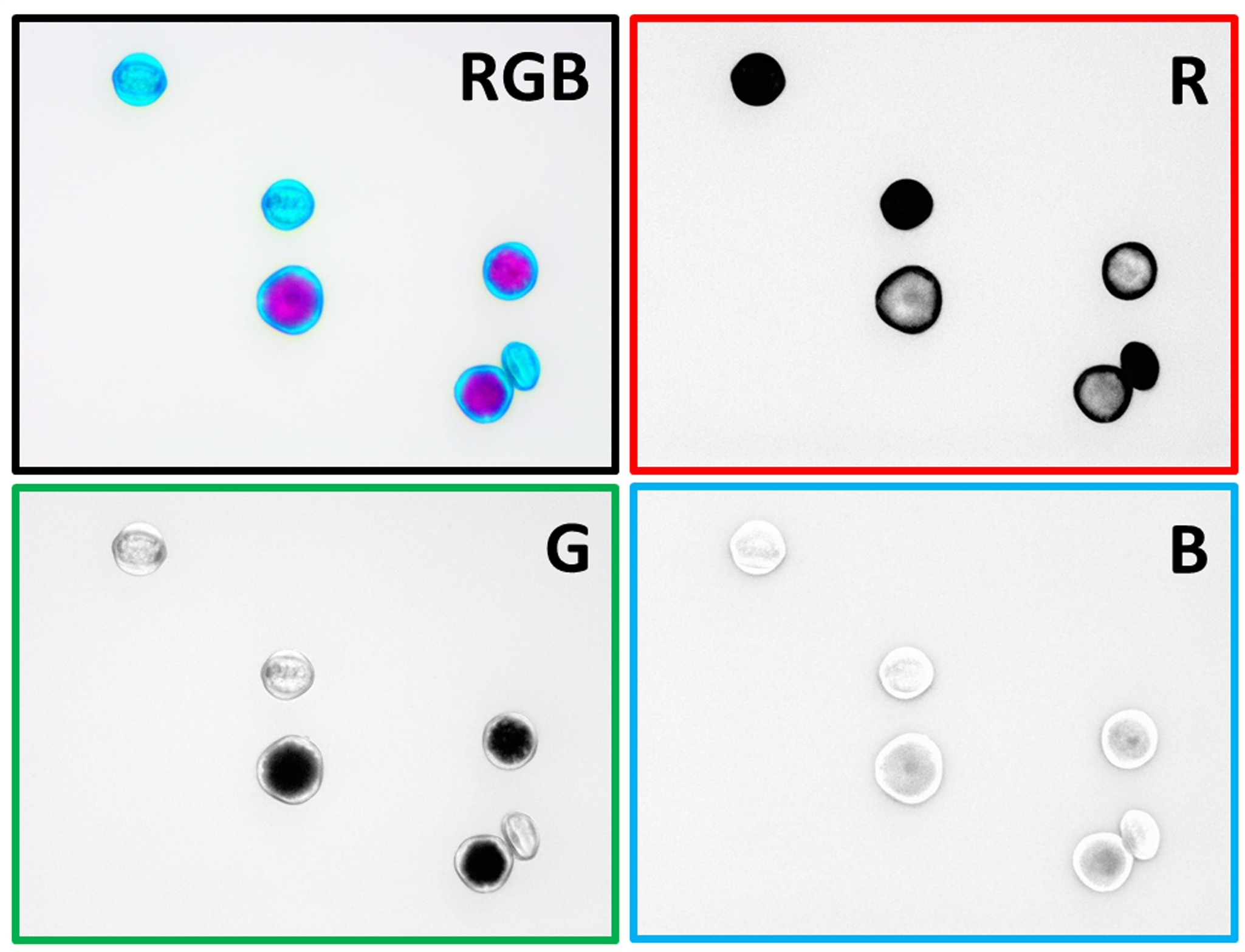

Supplement: Supplementary file 5 — Additional file 5. Separation of the red (R), green (G) and blue (B) layers of a RGB image of six grapevine pollen grains (three viable, three non-viable). To obtain red, green and blue color fractionated grayscale pictures, the original RGB image was processed using the “split channels” tool in Fiji. Pollen grains correspond to cv. Chardonnay. [file 13007_2017_267_MOESM5_ESM.tif]

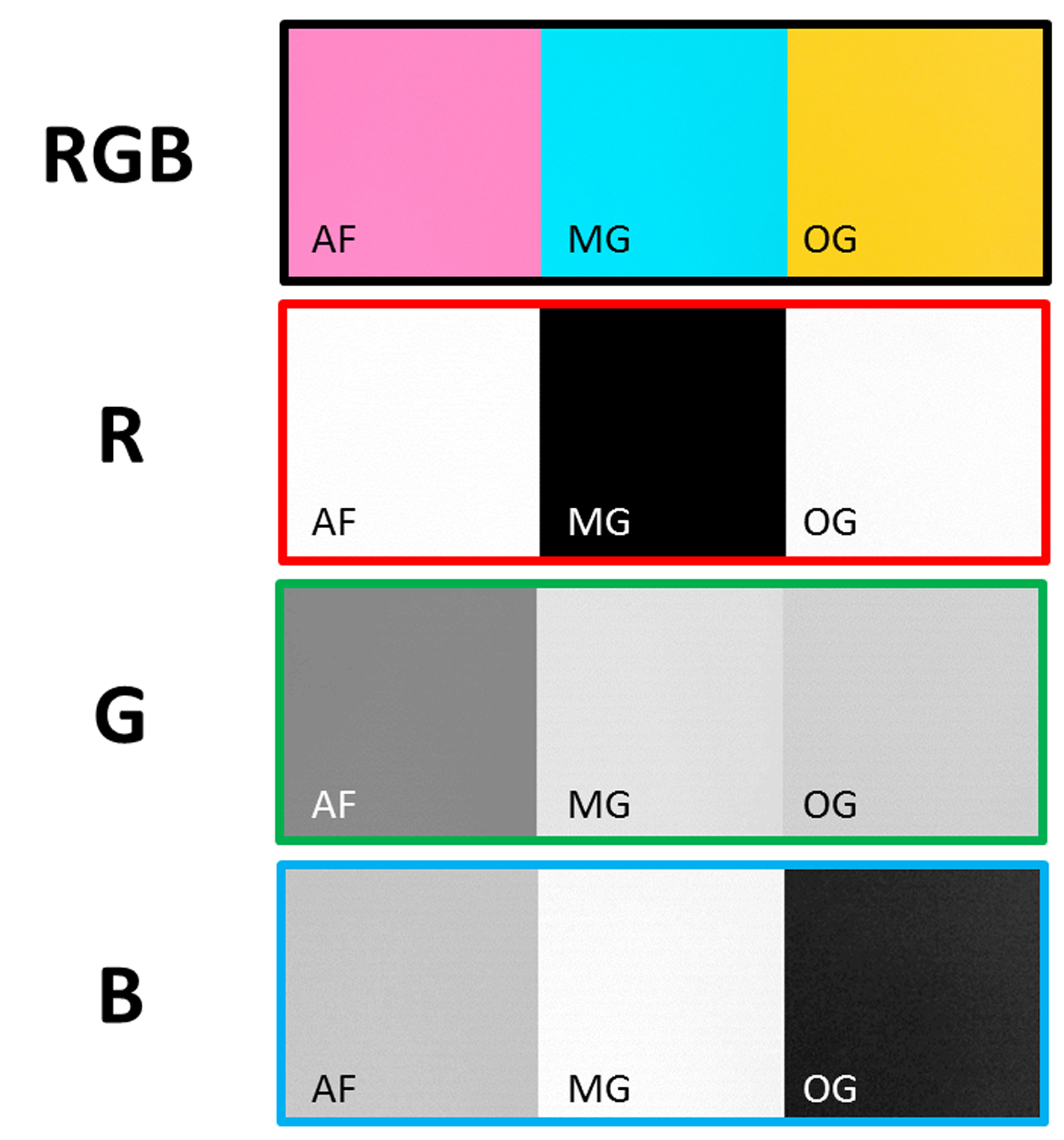

Supplement: Supplementary file 6 — Additional file 6. Separation of RGB images of the three dyes used in the Alexander’s modified staining solution (AF: Acid fuchsin; MG: Malachite green; OG: Orange G) on their red (R), green (G) and blue (B) basic layers. To obtain R, G and B color fractionated grayscale pictures, RGB images of pure dyes were processed using the “split channels” tool in Fiji. [file 13007_2017_267_MOESM6_ESM.tif]

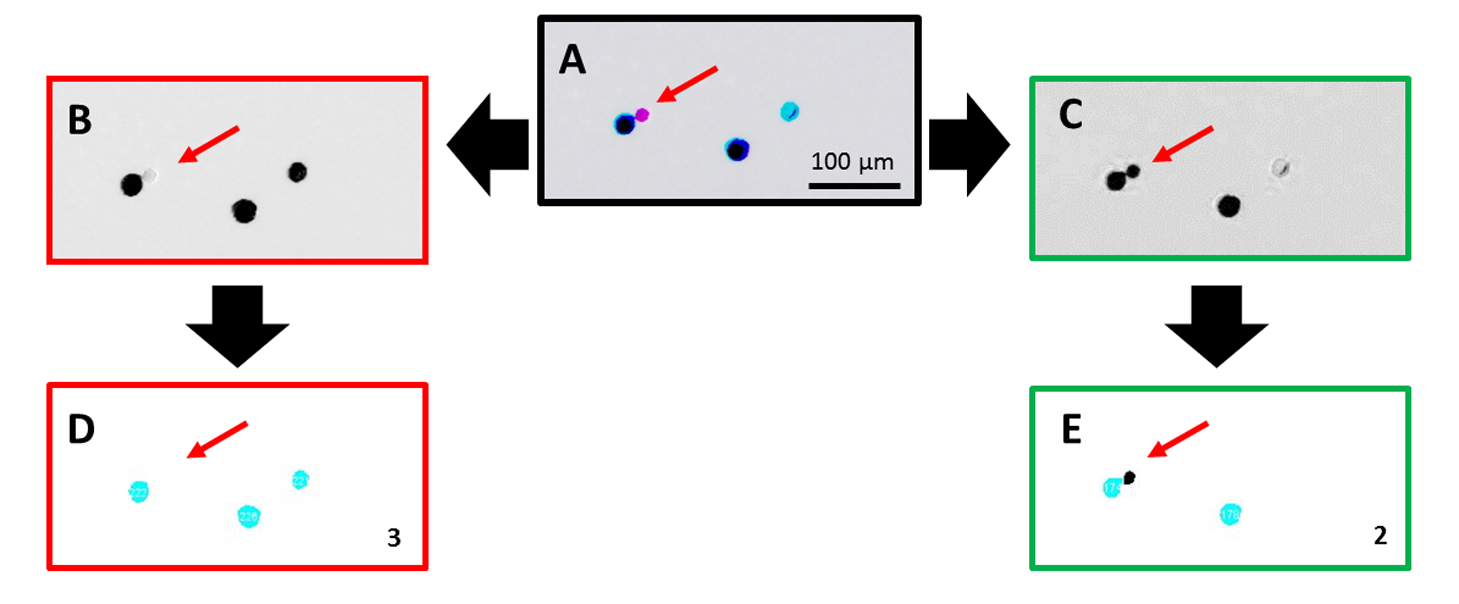

Supplement: Supplementary file 7 — Additional file 7. Original (A), red- and green-color fractionated grayscale pictures (B, C), and PollenCounter output images (D, E) obtained for a pollen sample of cv. Muscat Ottonel. An example of protoplasm content released from a viable pollen grain is indicated with a red arrow. Note that it is not detected in the red-channel derived images (B, D). Although detected in the green channel (C), the structure is not considered as a valid pollen grain to be counted (black region, E). For simplification, only a representative area of the processed image is shown. B and C images were obtained using the “split channels” tool in Fiji. The number of pollen grains automatically counted by PollenCounter (in cyan) is indicated in the lower right corners (D, E). [file 13007_2017_267_MOESM7_ESM.tif]

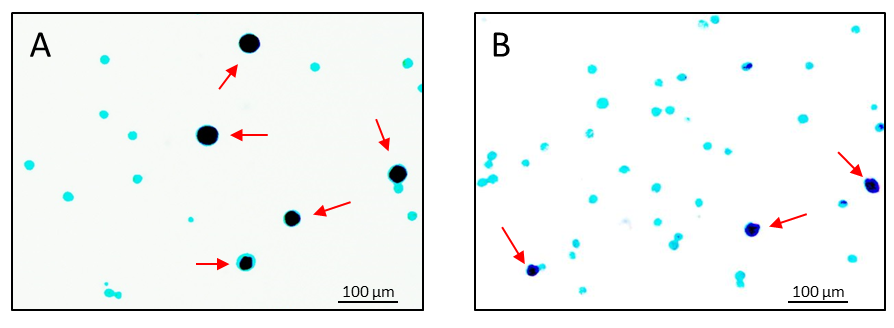

Supplement: Supplementary file 8 — Additional file 8. Corinto Bianco pollen grains after Alexander’s modified staining obtained in 2015 (A) and 2017 (B). Red arrows indicate viable pollen grains. For simplification, only representative areas are shown. [file 13007_2017_267_MOESM8_ESM.tif]

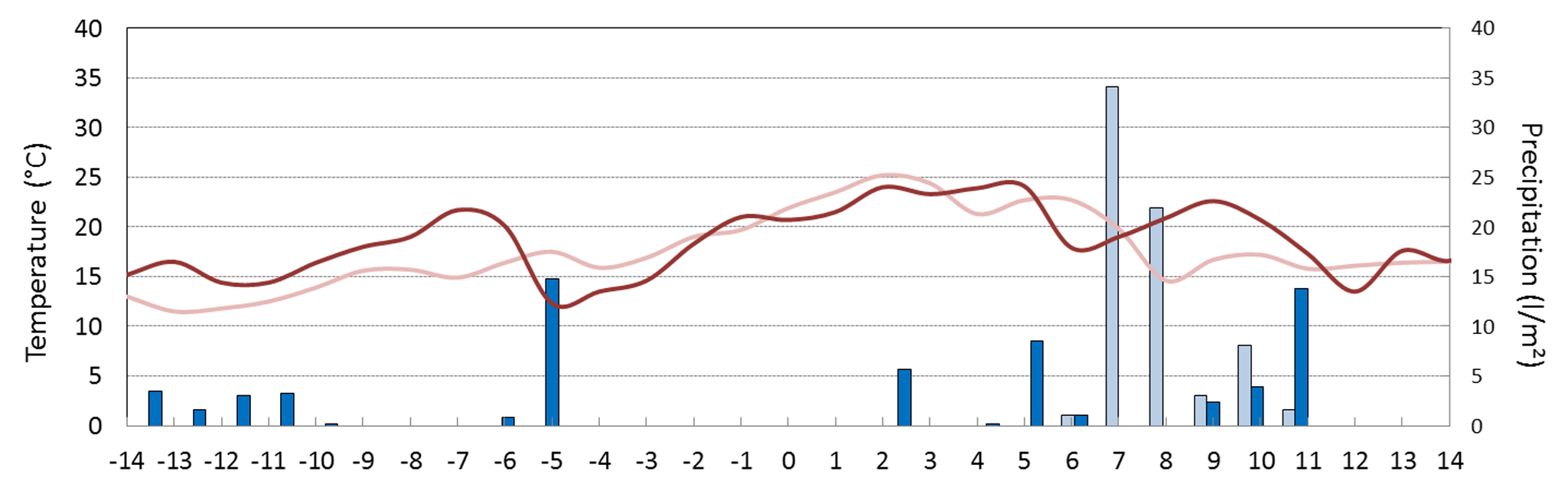

Supplement: Supplementary file 9 — Additional file 9. Pre- and post-flowering climate conditions in the Grapevine Germplasm Collection of the Instituto de Ciencias de la Vid y del Vino in 2015 and 2017. Days are shown according to the date when the first sample was collected in 2015 (2-June) and 2017 (23-May), indicated as a “0”. Sample collection extended for 14 and 10 days in 2015 and 2017, respectively. Light and dark red lines indicate mean temperatures for 2015 and 2017, respectively. Light and dark blue columns indicate accumulate daily rainfalls for 2015 and 2017, respectively. Data were obtained from La Rioja Government website (http://www.larioja.org/siar). [file 13007_2017_267_MOESM9_ESM.tif]
